# Synthesis of Alkyne-Substituted Dihydropyrrolones as Bacterial Quorum-Sensing Inhibitors of *Pseudomonas aeruginosa*

**DOI:** 10.3390/antibiotics11020151

**Published:** 2022-01-25

**Authors:** Basmah Almohaywi, Tsz Tin Yu, George Iskander, Shekh Sabir, Mohan Bhadbhade, David StC. Black, Naresh Kumar

**Affiliations:** 1 Department of Pharmaceutical Chemistry, College of Pharmacy, King Khalid University, Abha 6142, Saudi Arabia; bal-mohawe@kku.edu.sa; 2 School of Chemistry, The University of New South Wales, Sydney, NSW 2052, Australia; g.iskanderb@gmail.com (G.I.); s.sabir@student.unsw.edu.au (S.S.); d.black@unsw.edu.au (D.S.B.); 3 Solid State and Elemental Analysis Unit, Mark Wainwright Analytical Centre, Division of Research, The University of New South Wales, Sydney, NSW 2052, Australia; m.bhadbhade@unsw.edu.au

**Keywords:** quorum sensing, alkyne synthesis, *Pseudomonas aeruginosa*, dihydropyrrolones

## Abstract

The Quorum-sensing system in *Pseudomonas aeruginosa* is responsible for the pathogenicity and the production of virulence factors and biofilm formation. Dihydropyrrolones were previously found to act as inhibitors of QS-dependent bacterial phenotypes. In this study, a range of dihydropyrrolone (DHP) analogues was synthesized via the lactone-lactam conversion of lactone intermediates followed by the formation of novel acetylene analogues of dihydropyrrolones from brominated dihydropyrrolones via Sonogashira coupling reactions in moderate to high yields. Upon biological testing, the most potent compounds, **39**–**40** and **44,** showed higher bacterial quorum-sensing inhibitory (QSI) activity against *P. aeruginosa* reporter strain at 62.5 µM. Structure–activity relationship studies revealed that di-alkynyl substituent at the exocyclic position of DHPs possessed higher QSI activities than those of mono-alkynyl DHPs. Moreover, a hexyl-substituent at C3 of DHPs was beneficial to QSI activity while a phenyl substituent at C4 of DHPs was detrimental to QSI activity of analogues.

## 1. Introduction

*Pseudomonas aeruginosa* regulates its pathogenicity through an intercellular density-dependent communication system mediated by the binding of signaling molecules to quorum-sensing (QS) receptors such as LasR, which, upon its activation, modulates the expression of multiple genes responsible for the production of various virulence factors (e.g., pyocyanin, rhamnolipids, and pyoverdine), biofilm formation, the swarming motility, and antibiotic resistance. While conventional antibiotics exert increased selective pressure on bacteria, resulting in the development of antibiotic resistance [1,2,3], inhibition of quorum sensing interrupts the cell-to-cell coordination without exerting a selective pressure on bacteria. This may reduce the chance of bacterial resistance. Therefore, antagonists of LasR are considered an effective alternative strategy with a novel mode of actions for the treatment of infections while preventing bacterial resistance [1]. 

**Figure 1:**
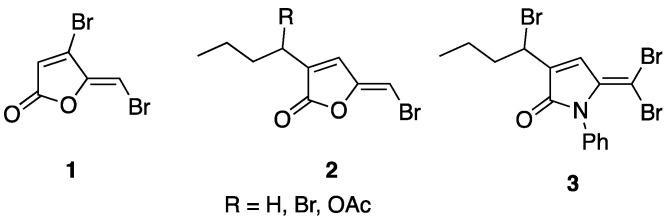
Furanones and DHP examples.

Fimbrolides, or halogenated furanones such as 5-dibrominated furanones **1** and **2** (Figure 1), were first synthesized in 1997 by Manny et al. [4] and are known to possess antimicrobial and quorum-sensing inhibitory (QSI) activities [5,6,7]. They were previously found to act as QS inhibitors of AHL that mediate QS phenotypes [5,6]. We previously reported the design, synthesis, and evaluation of fimbrolide–nitric oxide donor hybrids as antimicrobial agents [7]. However, their therapeutic use is limited by their instability, ease of hydrolysis, and toxicity [8]. Nevertheless, fimbrolides or 5-dibrominated furnaones can be utilized as a starting template for chemical manipulation into 1,5-dihydropyrrol-2-ones (DHPs) such as compound **3**. The dihydropyrrole-2-one moiety is present in several classes of biologically important natural and synthetic molecules such as rolipram, pulchellalactam [9], and Jatropham [10]. The 1,5-dihydropyrrol-2-one is an isosteric five-membered ring that is structurally related to furanones but contains a cyclic amide bond in substituting the hydrolytically and enzymatically labile cyclic ester in the natural and synthetic furanones. The lactam ring is hydrolytically more stable, which reduces the susceptibility of the ring-opening reaction by either enzymatically or chemical lactonolysis. [11,12] Several DHPs were found to possess antimicrobial activity, QS, and biofilm inhibitory activities [13] such as those reported by our group [14,15,16], including DHPs with a thioether [17] or a seleno-urea [18] moiety. In addition, the biological activities of DHP-related compounds were also studied by other research groups [19,20,21,22], including rubrolide derivatives, which showed antibacterial [23], anticancer [24], and herbicidal [25] activities. Moreover, a recent paper by Ma et al. also reported the synthesis and anti-virulence activities of several furanones and aryl-substituted pyrrolidone derivatives as quorum sensing inhibitors [26].

**Figure 2:**
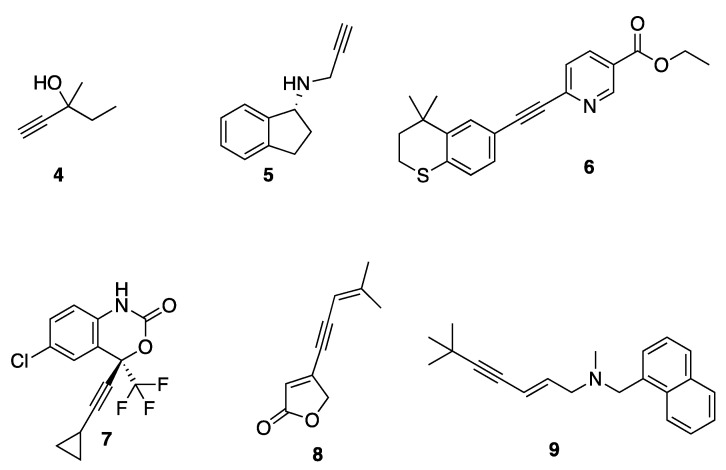
Examples of acetylene in pharmaceuticals.

The acetylene functionality is frequently found in many synthetic products as well as natural products that can be isolated from a wide range of plant species, fungal and bacterial cultures, and marine sponges and corals [27,28,29]. Acetylene groups are commonly used in medicinal chemistry for their electronic effects, equivalent to aromatic rings and providing structural rigidity (Figure 2) [30]. They are present in some of the sedative-hypnotic drugs such as meparfynol **4**, ethinamate, and the antiparkinsonian rasagiline **5** [30]. They are also present in the synthetic retinoid tazarotene **6**, which is used for the treatment of acne, psoriasis, and photo-aging, as well as the antiviral compound efavirenz **7**, the orally active contraceptives ethynyl-estradiol, norethindrone, and the implantable contraceptive etonogestrel [30]. Acetylene is also found in the natural pesticide falcarinol, the nervous system toxin oenanthotoxin, and the furanone-containing natural product cleviolide **8**. [31].

The installation of acetylene moieties into molecules can be achieved via the Sonogashira cross-coupling reaction between an organohalide and a terminal alkyne. The Sonogashira reaction was first reported in 1975 by Kenkichi Sonogashira and his co-workers. Since then, it has been one of the most important and commonly used cross-coupling reactions for forming carbon-carbon bonds. It is sometimes considered as important as the Suzuki–Miyaura reaction for the synthesis of compounds that are useful for medicinal chemistry research [32]. The Sonogashira cross-coupling reaction was featured in the synthesis of acetylenic derivatives of furanones [31,33] and quinolines with potential antimicrobial activity [34], as well as 2-aminoimidazole with antibiofilm activity [35]. It was also employed in the total synthesis of enediyne-containing antibiotics such as calicheamicin and dynemicin [36]. Many pharmaceutically important molecules were synthesized via the Sonogashira reaction, including terbinafine **9** (lamisil), which is used to treat fungal infections, and the aforementioned tazarotene **6**.

**Figure 3:**
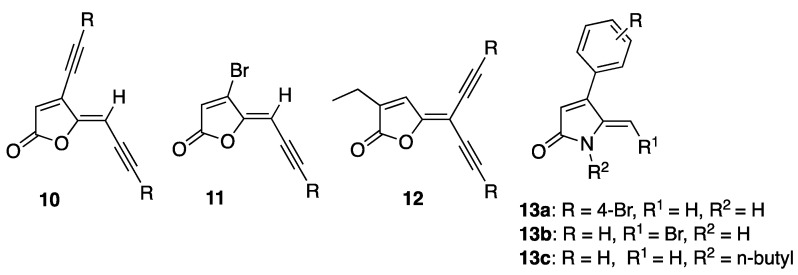
Acetylene-containing furanones based on our recent work.

Recently, our group reported the synthesis of acetylene-containing furanones **10**–**12**, which possessed low to moderate quorum-sensing inhibitory (QSI) activity, from brominated furanones **1** via Sonogashira cross-coupling reaction (Figure 3) [37]. In addition, we developed a method to convert brominated furanones into DHPs using a ring-opening/ring-closing lactamization reaction with amine nucleophiles [38]. We also reported the structural activity relationships (SAR) of our DHPs-based AHL mimics [15]. Dihydropyrrolones (DHPs) **13a**–**c** that were synthesized from their corresponding furanones using the ring-opening/ring-closing lactamisation reaction demonstrated good QSI and antibiofilm activities against *P. aeruginosa* [15] and *E. coli* [16] with minimal effect on bacterial growth [15,16]. Furthermore, our work on the synthesis of a series of thioether containing DHP analogues as PqsR antagonist and novel seleno- and thio-urea containing dihydropyrrol-2-one (DHP) analogues as LasR antagonists [18] was also reported. Prompted by these previous findings [14,15,18,37] and the possibility of exploiting the brominated exocyclic alkene at the 5-position of DHP, herein we aimed to introduce acetylene group(s) at the 5-position of the DHP and investigate their effect on the biological activity.

## 2. Results and Discussion

### 2.1. Synthesis

In this study, we investigated the introduction of acetylene substituents via the Sonogashira cross-coupling reaction of brominated DHPs to give acetylene-substituted DHPs. The reaction typically involves the use of a palladium catalyst and copper as a co-catalyst to form a carbon–carbon bond between a terminal alkyne, which serves as the coupling partner, and an aryl or vinyl halide [32,39].

**Scheme 1:**
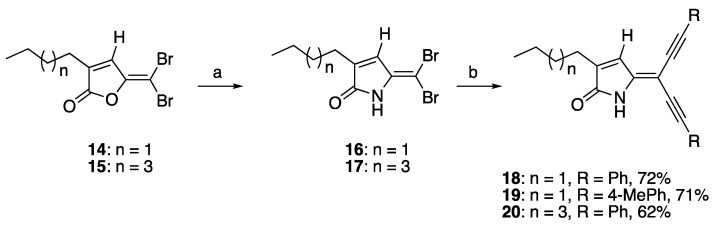
Lactamisation of furanone and Sonogashira coupling reaction of compounds **18**–**20.** Reaction conditions: (a) ammonia gas, DCM, r.t.; (b) PdCl_2_(PPh_3_)_2_ CuI, TEA, terminal alkyne, THF, reflux [40].

The synthesis of acetylene substituted DHPs was initially attempted by the lactamisation of the previously reported Sonogashira furanone products [37] with aqueous ammonia and/or alkylamine (e.g., propylamine) at room temperature and monitored by TLC. However, this synthetic strategy was proven to be unsuccessful. This could be attributed to the reduced reactivity of the acetylene-containing furanones towards lactamisation. Therefore, an alternative synthetic pathway for the synthesis of compounds **18**–**20** was sought. This alternative pathway involved the lactamisation of the brominated furanones **14**–**15** prior to the Sonogashira reaction. The lactamisation reaction of brominated furanones **14** and **15** using ammonia gas successfully afforded brominated DHPs **16** and **17**. The Sonogashira reaction was then carried out between the brominated DHPs **16**–**17** and phenylacetylene or 4-methylphenylacetylene (2.5 eq.) with the presence of CuI (0.1 eq.), bistriphenylphosphine)palladium(II) dichloride (PdCl_2_(PPh_3_)_2_ (0.1 eq.), and triethylamine (TEA, 1 eq.) in degassed THF under reflux conditions to give the di-alkynylated NH DHPs **18**–**20** in 62–72% yields as reported in our recently published conference paper (Scheme 1) [40]. During the optimization of rection process, it was found that heating was crucial for the success of the reaction and could minimize the formation of the acetylene homo-adduct. Moreover, it was found that the use of triethylamine as the solvent and the base could also result in the formation of the Sonogashira products, although the use of THF as the solvent in this reaction afforded the Sonogashira products in the highest yield [40].

Recently, we reported the QS inhibitory activities of 5-methylene-4-phenyl-1,5-dihydro-2*H*-pyrrol-2-ones such as compounds **21**–**23**, which can be synthesized via the lactone-lactam conversion method [15,41]. The synthesis began with the acid-catalyzed condensation of phenylacetones with glyoxylic acid, producing 5-hydroxyfuranones. In the key lactone-lactam conversion step, furanones were treated with thionyl chloride followed by aqueous ammonia to provide the intermediate 5-hydroxylactams, which were subsequently subjected to radical bromination with *N*-bromosuccinimide (NBS) to give 5-bromo-methylene-5-hydroxylactams, followed by dehydration to obtain the target compounds **21**–**23**.

**Scheme 2:**
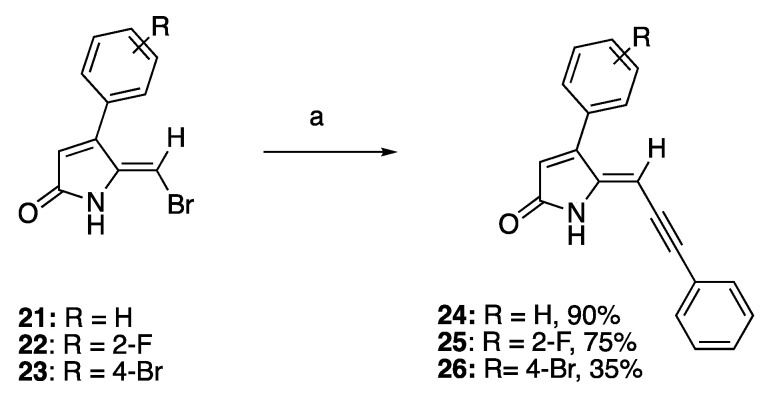
Sonogashira synthesis of 4-phenyl DHP derivatives. Reaction conditions: (a) PdCl_2_(PPh_3_)_2_, CuI, TEA, argon, alkyne, THF, and reflux.

To investigate the effect of introduction of a bulky mono-substituted acetylene group to the exocyclic vinylic alkene of compounds **21**–**23** while keeping the phenyl moiety on C4 on the QS activity of the compounds, 5-(bromomethylene) DHPs **21**–**23** were subjected to the Sonogashira coupling reaction with phenylacetylene, CuI, and palladium catalyst to generate compounds **24**–**26** in 35–90% yield (Scheme 2). While compounds **24** and **25** were synthesized in good yields of 90% and 75%, respectively, the low yield of compound **26** could be due to its poor solubility in organic solvent and high adsorption on silica gel, leading to considerable loss of the compound upon purification.

**Scheme 3:**
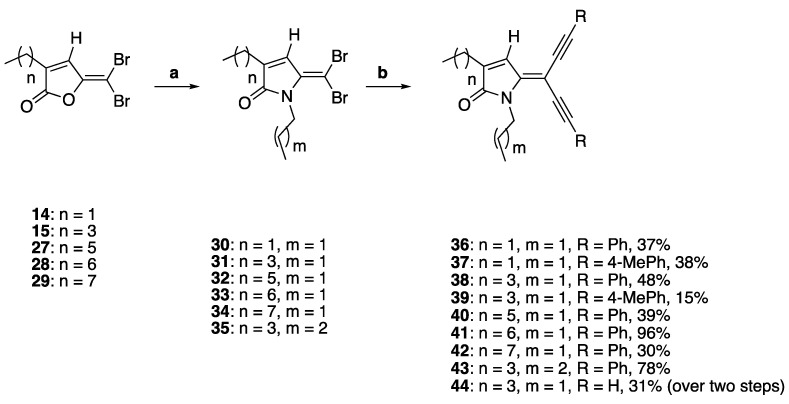
DHP analogues containing diacetylene groups and substituents at the ring nitrogen. Reaction conditions: (a) propylamine or butylamine, DCM, r.t., and 3 h; (b) PdCl_2_(PPh_3_)_2_, CuI, TEA, argon, alkyne, THF, and reflux.

To study the effect of introducing alkyl groups at the ring nitrogen, the synthesis of compounds **36**–**44** was investigated (Scheme 3). The precursor furanones **14**–**15** and **27**–**29** were synthesized following our previously reported procedure using sulfuric acid-catalyzed cyclization of brominated 2-alkyl-levulinic acids [4,7,42].

Following the synthesis of the precursor furanones **14**–**15** and **27**–**29**, the *N*-propyl analogues **30**–**34** were obtained in 30–96% yields by lactamization using excess equivalent of propylamine in dichloromethane, while the synthesis of **35** was synthesized using the same synthetic procedure for compounds **30**–**34** but with butylamine instead of propylamine [43]. The 5-dibromo DHPs **30** and **31** were reacted with phenylacetylene to give compounds **36** and **38** in 37% and 48% yield. Alternatively, the 5-dibromo DHPs **30** and **31** were also reacted with *p*-tolylacetylene to give alkyne compounds **37** and **39** in moderate to low yields of 37% and 15%, respectively. The longer chain 5-dibromo DHPs **40**–**43** were also synthesized from their corresponding 5-dibromo DHPs **30**–**35** in the analogous reactions in 30–96% yields. Finally, compound **44** with a terminal acetylene group was synthesized over two steps, by firstly reacting compound **31** with trimethylsilyl-protected acetylene followed by the deprotection of the trimethylsilyl protecting group to yield the terminal acetylene group.

The structures of the Sonogashira products **18**–**20**, **24**–**26,** and **36**–**44** were confirmed by spectroscopic analysis including ^1^H NMR, ^13^C NMR, and mass spectrometry (see Supplementary Materials File S1). In the acetylene products, the C4 hydrogen resonance of **18**–**20** and **36**–**44** was observed to shift downfield in the Sonogashira coupling products at 7.13–7.26 ppm compared to 7.02 ppm in the DHP intermediates. The additional phenyl ring proton resonances for compounds **18**–**20** and **36**–**44** were observed at 7.20–7.60 ppm in the aromatic region of the ^1^H NMR spectra. Meanwhile, the newly formed acetylene carbons were confirmed by ^13^C NMR and were observed at 83.0–98.0 ppm. The number of aliphatic carbons was also confirmed by ^13^C NMR for compounds **18**–**20** and **36**–**44** as these carbons resonated in the aliphatic region between 22.0 and 40.0 ppm. The CH_2_ adjacent to the ring nitrogen was observed at 30.0–40.0 ppm in the ^13^C NMR spectrum. The exocyclic singlet protons (C=CHBr) adjacent to the newly formed acetylene phenyl of the Sonogashira products **24**–**26** were observed upfield at 5.47–5.59 ppm compared to 6.16–6.35 ppm in the DHP intermediates **21**–**23**.

### 2.2. X-ray Analysis of Sonogashira DHP Compounds

**Figure 4:**
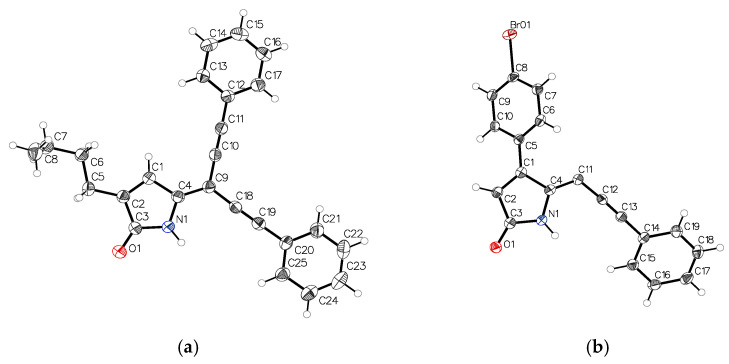
ORTEP representation of compounds **18** (**a**) and **26** (**b**).

To confirm the structures of the acetylene compounds **18** (C3-butyl, C4-H) and **26** (C4-(*p*-Br-phenyl), these compounds were crystallized from acetonitrile to give yellow needles and yellow plate-shaped crystals, respectively, that were suitable for single crystal X-ray diffraction. The ORTEP views of molecules **18** and **26** along with the labelling of atoms are presented in Figure 4a,b.

**Figure 5:**
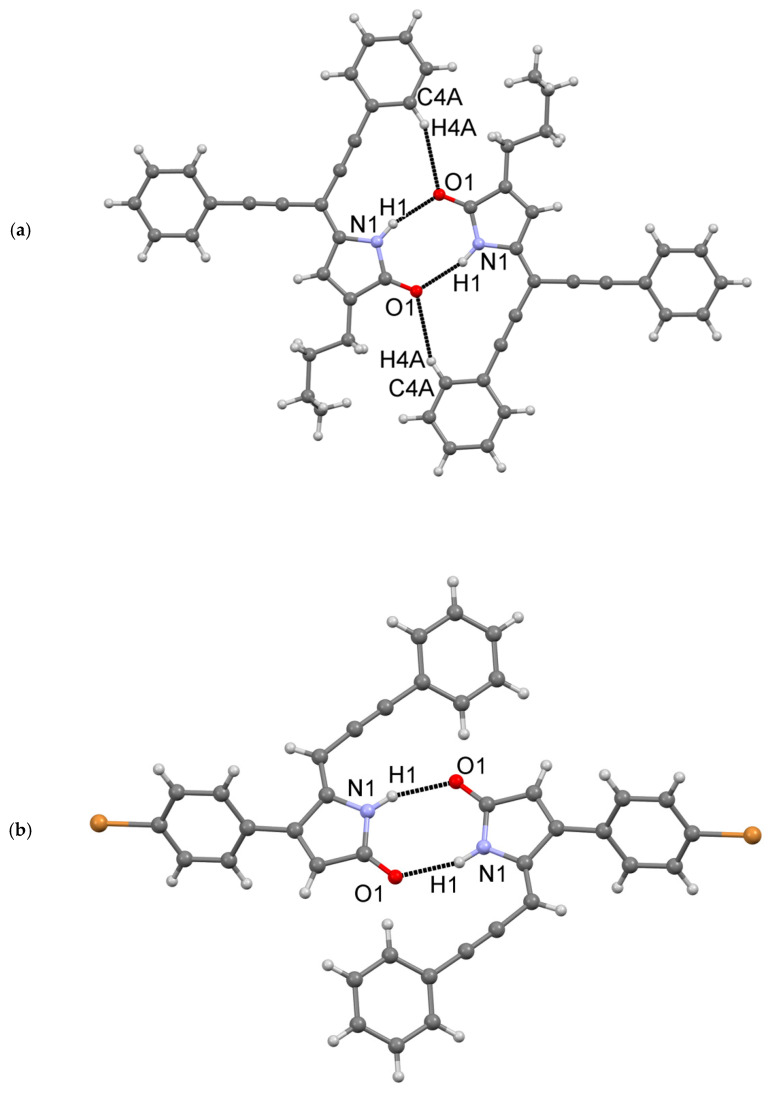
Dimeric association via H-bonding interactions in structures of compounds **18** (**a**) and (**b**) **26**.

Compound **18** with a butyl group at C3 is essentially planar, whereas molecule **26** with a *p*-bromophenyl group at C4 is not planar. In **26**, the phenyl ring C5-C10 is rotated about the C1-C5 bond by −54.3°, presumably to avoid the short H…H contact of the hydrogens from carbon atoms C6 and C11. Both **18** (Figure 5a) and **26** (Figure 5b) self-associate in a very similar fashion via N…O hydrogen bonds. An additional C-H…O interaction (“hydrogen bond”) was also observed in **18**.

### 2.3. QS Inhibition

QS inhibition assay was performed on the synthesized acetylene DHPs to evaluate their efficacy and to study their structure–activity relationship (SAR). These compounds were tested against *P. aeruginosa* MH602, a reporter strain that measures the level of green fluorescent protein (GFP), following the method reported by Hentzer et al. [5] An inhibitor is expected to reduce the expression and the production of GFP. In this study, the liquid cultures of the *P. aeruginosa* reporter strain MH602 were incubated in various concentrations (250, 125, and 62.5 µM) of the synthesized compounds and the fluorescence of GFP at λ = 535 nm was recorded. A fimbrolide, furanone 30 (**1**), was used as a positive control to validate the assay protocol. The optical density (OD) at 600 nm was also measured to determine the potential effect of the tested compounds on bacterial growth to ensure that their QSI activity was not due to bactericidal effects.

The results for the tested compounds are presented in Table 1. The percentage QS inhibition of the compounds was calculated as the percentage difference of GFP intensity between the sample and the control at the same time point when the fluorescence reached its maximum value in the control. The acetylene compounds reduced QS by 43–80% at 250 µM, 28–66% at 125 µM, and 24–56% at 62.5 µM. Moreover, most of the alkyne derivatives did not have a substantial effect on the viability of bacterial cells.

Among the di-alkynyl DHPs **18**–**20** with a C3 alkyl group but no substituent at N1-position, molecules **18** and **20** bearing a terminal phenyl ring possessed higher QS inhibition at 62.5 µM (QSI = 47.8% and 39.3%, respectively) than compound **19** bearing a *p*-methylphenyl group (QSI = 24.1%). Interestingly, while the installation of the phenylacetylene groups generally increased the QSI activities of the analogues, the introduction of a methyl-substituent at the terminal phenyl rings decreased the QSI activity significantly. DHP **19** with a methyl-substituent at the terminal phenyl rings had a significantly lower QSI activity of 24.1% when compared to its parent compounds **18** (QSI = 47.8%) with unsubstituted terminal phenyl rings and **16** (QSI = 44.7%) without the phenylacetylene moiety at 62.5 µM.

On the other hand, the *N*-unsubstituted 4-aryl DHPs **24** (C4-phenyl), **25** (C4-2-fluorophenyl), and **26** (C4-4-bromophenyl) with a single acetylene unit at the exocyclic position showed lower QS inhibition compared to their parent molecules **21**–**23** without the acetylene substituents [15]. The observed reduction in QS activity could be attributed to the bulky phenyl and acetylene groups, which hinder the molecules from binding to the bacterial receptor protein. This observation is consistent with lower activities of the mono- and di-substituted acetylene-derived furanones reported by Biswas et al. [37].

Among the di-alkynyl DHPs bearing aliphatic chains at both C3 and N1, compound **44** with unsubstituted terminal acetylene moieties possessed the highest QSI activities at all tested concentrations. The introduction of a terminal phenyl ring was detrimental to QSI activity as the corresponding compound **38** with a terminal phenyl ring at the acetylene moieties showed a lower QSI of 40.6% compared to compound **44** with a QSI of 55.8% at 62.5 µM. Interestingly, the effect of the introduction of an alkyl group at N1-position of the DHP on the QSI activity of the analogues depends on the substituent at the terminal phenyl rings of the acetylene moieties. For compounds with a phenylacetylene moiety, the introduction of a methyl-substituent at N1 position decreased the QSI activity slightly as *N*-methyl-substituted compound **38** possessed a lower QSI of 40.6% compared to its corresponding unsubstituted compound **18** (QSI = 47.8%) at 62.5 µM. In contrast, the introduction of a methyl-substituent at N1 position of DHP bearing a *p*-methylphenylacetylene moiety enhanced the QSI activity of the analogue significantly as *N*-methyl-substituted compound **39** possessed a higher QSI of 54.1% compared to its corresponding unsubstituted compound **19** (QSI = 24.1%) at 62.5 µM. In addition, lengthening the *N*-methyl-substituent to *N*-ethyl-substituent was found to be detrimental to QSI activity as *N*-ethyl-substituted compound **43** exhibited a lower QSI of 24.3% compared to the corresponding *N*-methyl-substituted compound **38**, which exhibited a QSI of 40.6% at 62.5 µM. Moreover, compounds with different aliphatic chain length at C3 were also synthesized to investigate the optimum chain length for the highest QSI activity. It was found that a hexyl-substituent at C3 was optimum for QSI activity, in which compound **40** with a hexyl-substituent at C3 possessed the highest QSI of 52.9% at 62.5 µM among this series of compounds. Compounds with a longer or shorter alkyl chain length generally showed lower QSI activities.

## 3. Experimental

### 3.1. Quorum-Sensing Inhibition Assay for PAMH602

The *P. aeruginosa* MH602 PlasB::gfp (ASV) reporter strain was used. An overnight culture was prepared in Luria–Bertani (LB10) media supplemented with gentamycin (40 μM). This bacterial culture solution was diluted (1 in 100) with LB10 supplemented with gentamycin (15 μM). Stock solutions of the synthesized compounds were prepared at 20 mM in DMSO. Compounds were pipetted into each well with final concentrations of 250, 125, and 62.5 μM (in triplicate) with a final volume of 200 μL with the prepared bacterial culture. The negative control was prepared containing 200 μL of the bacterial culture without the tested compounds. The plates were incubated at 37 °C for 15 h. The plates were measured for GFP expression (fluorescence: excitation 485 nm, emission 535 nm) using a microplate reader (Wallac Victor, Perkin-Elmer, Minneapolis-Saint Paul, MN, USA), and the cell growth was also assessed by recording the OD at 600 nm.

### 3.2. Methodology for X-ray Crystallography

A suitable single crystal of **18** (CCDC 2131315) and **26** (CCDC 2131273) obtained from the recrystallisation of the corresponding compound from acetonitrile was selected under a polarizing microscope (Leica M165Z) mounted on a MicroMount (MiTeGen, Ithaca, NY, USA) consisting of a thin polymer tip with a wicking aperture. The X-ray diffraction measurements were carried out on a Bruker D8 Quest Single Crystal diffractometer with Photon II detector at 150 K by using IμS 3.0 Microfocus Source with Mo-K*α* radiation (λ = 0.710723 Å). The single crystal, mounted on the goniometer using cryo loops for intensity measurements, was coated with paraffin oil and then quickly transferred to the cold stream using an Oxford Cryo stream 800 attachment. Symmetry-related absorption corrections using the program SADABS were applied and the data were corrected for Lorentz and polarization effects using Bruker APEX3 software [44]. The structure was solved by ShelxT (intrinsic phasing) [45] and the full-matrix least-square refinement was carried out using Shelxl [46] in Olex2 [47]. The non-hydrogen atoms were refined anisotropically. The molecular graphic was generated using program Olex2 [47] (Supplementary Materials Tables S3 and S4).

Crystal data: compound **18** C_25_H_21_NO (Table S1), M.W. = 351.4490, Monoclinic. Cell dimensions: a = 8.8068 (7) Å, b = 20.8376 (15) Å, c =10.6832 (6), and beta = 104.884 (2) Å; compound **26** C_19_H_12_BrNO (Table S2), M.W. = 350.2150, Monoclinic, Cell dimensions: a = 11.944 (4) Å, b = 9.578 (3) Å, c =13.798 (4), and beta = 105.385 (6) Å. Crystallographic data have been deposited with the Cambridge Crystallographic Data Centre with publication numbers CCDC 2131315 and CCDC 2131273. A copy of the data can be obtained free of charge from CCDC, 12 Union Road, Cambridge, CB2 1EZ, UK, or by e-mail: deposit@ccdc.cam.ac.uk.

### 3.3. Synthesis Procedures

#### 3.3.1. General Information

Commercially available reagents were purchased from standard suppliers such as Sigma Aldrich, Alfa Aesar, Combi-Blocks, and Oakwood Chemicals. The synthetic procedures have been reported for all compounds as general methods and appropriate references have been given for known compounds. Melting points were measured using an OptiMelt melting point apparatus and are reported without correction. High-resolution mass spectra were recorded by the Bioanalytical Mass Spectrometry Facility, UNSW, on an Orbitrap LTQ XL ion trap mass spectrometer using a nanospray (nano-electrospray) ionization source under positive ESI mode. The ^1^H and ^13^C NMR spectra were determined in the designated solvent on a Bruker DPX 300 spectrometer or a Bruker Avance 400 spectrometer. Chemical shifts (δ) are quoted in parts per million (ppm) internally referenced relative to the solvent nuclei. Multiplicities in ^1^H NMR are assigned as follows: brs, broad singlet; s, singlet; d, doublet; t, triplet; q, quartet; quint, quintet; sext, sextet; m, multiplet; or as a combination (e.g., dd, dt, td, etc.). The coupling constant (*J*) in hertz, integration, and proton count was also reported.

#### 3.3.2. Synthesis of Brominated DHPs’ Intermediates **30–35**

The 5-Br DHPs were synthesized from furanones **14**–**15** and **27**–**29**. Furanones (1 mmol) were dissolved in DCM (5 mL) and the solution was cooled in an ice bath before the addition of propylamine (4 eq., 4 mmol), and then the reaction mixture was left to stir for 2 h. The solvent was evaporated and the oil residue was dehydrated with TFA (1 eq.) to give intermediates **30** to **34,** which were purified by flash chromatography with 50% dichloromethane in hexane to give intermediate DHPs **30** to **34**. Synthetic procedures were reported of other intermediates **16**, **17,** and **35** previously [7,43].

#### 3.3.3. Sonogashira Coupling Reaction Procedure

A mixture of acetylene (2.5 eq.) and TEA (1 eq.) in THF was purged with argon or nitrogen for 20 min before the addition of the 5-bromo DHPs (1 mmol), CuI (0.1 eq.), and PdCl_2_(PPh_3_)_2_ (0.1 eq.), and the mixture was heated at 60 °C for 18 h. The THF in the reaction mixture was evaporated, redissolved in DCM, washed with 2 M HCl (5 mL × 2), and the organic layer was dried over sodium sulphate. The crude mixtures were purified by flash chromatography using gradient solvent mixture of dichloromethane and hexane (10 to 50% gradient).

### 3.4. Compounds’ Full Characterizations

**5-(Dibromomethylene)-3-ethyl-1-propyl-1,5-dihydro-2*H*-pyrrol-2-one (30)**

Yellow semi-solid (30%); ^1^H NMR (CDCl_3_, 400 MHz): δ 0.91 (t, *J* = 8.0 Hz, 3H, CH_3_), 1.20 (t, *J* = 8.0 Hz, 3H, CH_3_), 1.61–1.70 (m, 2H, CH_2_), 2.34–2.38 (m, 2H, CH_2_), 3.93–3.97 (m, 2H, CH_2_), 7.02 (s, 1H, C4-H); ^13^C NMR (CDCl_3_, 100 MHz): δ 10.8 (CH_3_), 11.7 (CH_3_), 18.9 (CH_2_), 23.4 (CH_2_), 42.3 (CH_2_), 73.5 (C), 131.3 (CH), 140.2 (C), 140.7 (C), 171.9 (C=O); IR (ATR): ν_max_ 753, 878, 1044, 1451, 1688, 2925, 2967 cm^−1^; UV-VIS (MeOH): λ_max_ 285 nm (ε 13,859 cm^−1^M^−1^); HRMS (C_10_H_13_^79^Br_2_NO) calcd m/z 343.9256 [M + Na]^+^, obsd m/z 343.9258 [M + Na]^+^ and HRMS (C_10_H_13_^81^Br_2_NO) calcd m/z 347.9215 [M + Na]^+^, obsd m/z 349.9215 [M + Na]^+^.

**3-Butyl-5-(Dibromomethylene)-1-propyl-1,5-dihydro-2*H*-pyrrol-2-one (31)**

Yellow semi-solid (61%); ^1^H NMR (CDCl_3_, 400 MHz): δ 0.94–0.98 (m, 6H, CH_3_ × 2), 1.28–1.44 (m, 2H, CH_2_), 1.54–1.70 (m, 6H, CH_2_ × 3), 2.32–2.36 (m, 2H, CH_2_), 3.93–3.95 (m, 2H, CH_2_) 7.02 (s, 1H, C4-H); ^13^C NMR (CDCl_3_, 100 MHz): δ 10.8 (CH_3_), 13.8 (CH_3_), 22.4 (CH_2_), 23.4 (CH_2_), 25.2 (CH_2_), 29.6 (CH_2_), 42.3 (CH_2_), 73.1(C), 131.8 (CH), 138.8 (C), 140.7 (C), 172.1 (C); IR (ATR): ν_max_ 694, 851, 1188, 1345, 1508, 1709, 3171 cm^-−1^; UV-VIS (MeOH): λ_max_ 285 nm (ε 13,920 cm^−1^M^−1^); HRMS (C_12_H_17_^79^Br_2_NO) calcd m/z 349.9750 [M + H]^+^, obsd m/z 349.9751 [M + H]^+^ and HRMS (C_12_H_17_^81^Br_2_NO) calcd m/z 353.9709 [M + H]^+^, obsd m/z 353.9709 [M + H]^+^.

**5-(Dibromomethylene)-3-hexyl-1-propyl-1,5-dihydro-2*H*-pyrrol-2-one (32)**

Yellow semi-solid (58%); ^1^H NMR (CDCl_3_, 400 MHz): δ 0.91 (t, *J* = 8.0 Hz, 6H, CH_3_ × 2), 1.30–1.40 (m, 6H, CH_2_ × 3), 1.54–1.68 (m, 4H, CH_2_ × 2), 2.31–2.35 (m, 2H, CH_2_), 3.93–3.97 (m, 2H, CH_2_), 7.02 (s, C4-H); ^13^C NMR (CDCl_3_, 100 MHz): δ 10.9 (CH_3_), 14.1 (CH_3_), 22.5 (CH_2_), 23.4 (CH_2_), 25.5 (CH2), 27.5 (CH_2_), 28.9 (CH_2_), 31.5 (CH_2_), 42.3 (CH_2_), 70.5 (C), 131.8 (CH), 138.9 (C), 140.7 (C), 172.1 (C=O); IR (ATR): ν_max_ 753, 816, 1172, 1440, 1594, 1694, 2923 cm^−1^; UV-VIS (MeOH): λ_max_ 285 nm (ε 53,601 cm^−1^M^−1^); HRMS (C_14_H_21_Br_2_NO) calcd m/z 378.0063 [M + H]^+^, obsd m/z 378.0065 [M + H]^+^ and HRMS (C_14_H_21_^81^Br_2_NO) calcd m/z 382.0022 [M + H]^+^, obsd m/z 382.0023 [M + H]^+^.

**5-(Dibromomethylene)-3-heptyl-1-propyl-1,5-dihydro-2*H*-pyrrol-2-one (33)**

Yellow semi-solid (87%); ^1^H NMR (CDCl_3_, 400 MHz): δ 0.90–0.93 (m, 6H, CH_3_), 1.28–1.36 (m, 10H, CH_2_), 1.61–1.66 (m, 2H, CH_2_) 2.31–2.36 (m, 2H, CH_2_), 3.95 (t, *J* = 8.0 Hz, CH_2_), 7.02 (s, 1H, CH); ^13^C NMR (CDCl_3_, 100 MHz): δ 10.9 (CH_3_), 14.1 (CH_3_), 22.6 (CH_2_), 23.4 (CH_2_), 25.5 (CH_2_), 27.5 (CH_2_), 28.9 (CH_2_), 29.3 (CH_2_), 31.7 (CH_2_), 42.3 (CH_2_), 73.5 (C); UV-VIS (MeOH): λ_max_ 285 nm (ε 56,642 cm^−1^M^−1^); HRMS (C_15_H_23_Br_2_NO) calcd m/z 392.0219 [M + H]^+^, obsd m/z 392.0219 [M + H]^+^ and HRMS (C_15_H_23_^81^Br_2_NO) calcd m/z 396.0178 [M + H]^+^, obsd m/z 396.0175 [M + H]^+^.

**5-(Dibromomethylene)-3-octyl-1-propyl-1,5-dihydro-2*H*-pyrrol-2-one (34)**

Yellow semi-solid (68%); ^1^H NMR (CDCl_3_, 400 MHz): δ 0.90–0.97 (m, 6H, CH_3_), 1.90–1.93 (m, 12H, CH_2_), 1.57–2.35 (m, CH_2_), 2.33 (t, *J* = 8.0 Hz, 2H, CH_2_), 3.95 (t, *J* = 8.0 Hz, 2H, CH_2_), 7.03 (s, 1H, CH); ^13^C NMR (CDCl_3_, 100 MHz): δ 10.9 (CH_3_), 14.1 (CH_3_), 22.7 (CH_2_), 23.4 (CH_2_), 25.5 (CH_2_), 27.5 (CH_2_), 29.2 (CH_2_), 29.3 (CH_2_), 31.8 (CH_2_), 42.4 (CH_2_), 73.9 (C), 131.9 (CH), 138.8 (C), 140.7 (C), 172.2 (C=O); IR (ATR): ν_max_ 751, 824, 1132, 1197, 1459, 1687, 2922 cm^−1^; UV-VIS (MeOH): λ_max_ 285 nm (ε 15,452 cm^−1^M^−1^); HRMS (C_16_H_25_^79^Br_2_NO) calcd m/z 406.0376 [M + H]^+^, obsd m/z 406.0380 [M + H]^+^ and HRMS (C_16_H_25_^81^Br_2_NO) calcd m/z 410.0335 [M + H]^+^, obsd m/z 410.0338 [M + H]^+^.

#### DHP Acetylene Analogues

**3-Butyl-5-(1,5-diphenylpenta-1,4-diyn-3-ylidene)-1,5-dihydro-2*H*-pyrrol-2-one (18)**

Yellow solid (72%); m.p. 215 °C; ^1^H NMR (CDCl_3_, 400 MHz): δ 0.93 (t, *J* = 8.0 Hz, 3H, CH_3_), 1.38–1.48 (m, 2H, CH_3_), 1.58–1.61 (m, 2H, CH_2_), 2.40–2.42 (q, *J* = 8.0 Hz, 2H, CH_2_), 7.11 (s, 1H, C4-H), 7.26–7.38 (m, 6H, ArH), 7.53–7.57 (m, 4H, ArH), 8.06 (s, NH); ^13^C NMR (CDCl_3_, 100 MHz): δ 13.9 (CH_3_), 22.5 (CH_2_), 25.4 (CH_2_), 29.9 (CH_2_), 65.9 (=C-Br_2_), 83.8 (C), 86.9 (C), 93.5 (C), 97.5 (C), 122.1 (C), 122.5 (C), 128.0 (C), 128.4 (CH), 128.5 (CH), 128.8 (C), 129.2 (C), 131.6 (CH), 131.7 (CH), 141.1 (C), 148.6 (C), 170.6 (C=O); IR (ATR): ν_max_ 752, 848, 1124, 1486, 1685, 3155 cm^−1^; UV-VIS (MeOH): λ_max_ 390 nm (ε 12,962 cm^−1^M^−1^) 295 (10,741); HRMS (C_25_H_21_NO) calcd m/z 352.1696 [M + H]^+^, obsd m/z 352.1695 [M + H]^+^.

**3-butyl-5-(1,5-di-p-tolylpenta-1,4-diyn-3-ylidene)-1,5-dihydro-2*H*-pyrrol-2-one (19)**

Yellow solid (71%); m.p. 148 °C; ^1^H NMR (CDCl_3_, 400 MHz): δ 0.97 (t, *J* = 8.0 Hz, 3H, CH_3_), 1.41–1.46 (m, 2H, CH_2_), 1.59–1.65 (m, 4H, CH_2_), 2.42–2.46 (m, 2H, CH_2_), 7.13 (s, 1H, C4-H), 7.19–7.21 (m, 4H, ArH), 7.28–7.46 (m, 4H, ArH), 7.87 (s, NH); ^13^C NMR (CDCl_3_, 100 MHz): δ 13.8 (CH_3_), 21.6 (CH_2_), 22.5 (CH_2_), 25.4 (CH_2_), 30.0 (CH_2_), 83.5 (=C-Br_2_), 87.4 (C), 87.4 (C), 93.7 (C), 97.8 (C), 119.0 (C), 128.0 (C), 129.2 (CH), 131.5 (CH), 139.1 (C), 139.5 (C), 141.8 (C), 148.0 (C), 170.5 (C=O); IR (ATR): ν_max_ 753, 812, 1090, 1508, 1685, 2959 cm^−1^; UV-VIS (MeOH): λ_max_ 390 nm (ε 5560 cm^−1^M^−1^) 300 (5370); HRMS (C_27_H_25_NO) calcd m/z 380.2009 [M + H]^+^, obsd m/z 380.2009 [M + H]^+^.

**5-(1,5-Diphenylpenta-1,4-diyn-3-ylidene)-3-hexyl-1,5-dihydro-2*H*-pyrrol-2-one (20)**

Yellow solid (62%); m.p. 156 °C; ^1^H NMR (CDCl_3_, 400 MHz): δ 0.92 (t, *J* = 8.0 Hz, 3H, CH_3_), 1.28–1.40 (m, 6H, CH_2_), 1.42–1.66 (m, 2H, CH_2_), 2.42–2.46 (m, 2H, CH_2_), 7.14 (s, 1H, C4-H), 7.28–7.41 (m, 6H, ArH), 7.56–7.60 (m, 4H, ArH), 8.11 (s, NH); ^13^C NMR (CDCl_3_, 100 MHz): δ 14.1 (CH_3_), 22.5 (CH_2_), 25.7 (CH_2_), 27.9 (CH_2_), 29.1 (CH_2_), 31.6 (CH_2_), 83.8 (C), 86.9 (C), 93.5 (C), 97.5 (C), 122.1 (C), 128.0 (C), 128.5 (CH), 128.9 (C), 129.2 (C), 131.6 (CH), 141.1 (C), 148.6 (C), 170.6 (C=O); IR (ATR): ν_max_ 752, 845, 1096, 1370, 1488, 1684, 2918 cm^–1^; UV-VIS (MeOH): λ_max_ 390 nm (ε 5655 cm^−1^M^−1^), 295 (4934); HRMS (C_27_H_25_NO) calcd m/z 380.2009 [M + H]^+^, obsd m/z 380.2010 [M + H]^+^.

**(*Z*)-4-Phenyl-5-(3-phenylprop-2-yn-1-ylidene)-1,5-dihydro-2*H*-pyrrol-2-one (24)**

Brown semi-solid (90%); ^1^H NMR (CDCl_3_, 400 MHz): δ 5.59 (s, 1H, CH), 6.25 (s, 1H, C3-H), 7.37–7.47 (m, 3H, ArH), 7.48–7.56 (m, 7H, ArH), 8.30 (s, 1H, NH); ^13^C NMR (CDCl_3_, 100 MHz): δ 84.9 (C), 92.9 (C), 101.4 (C), 121.6 (ArC), 122.5 (CH), 128.5 (ArCH × 2), 125.6 (ArCH), 128.9 (ArCH × 2), 129.1 (ArCH), 129.7 (ArCH), 131.3 (ArC), 131.7 (ArCH), 145.9 (C), 150.5 (C), 169.5 (C=O); IR (ATR): ν_max_ 691, 758, 1682, 3140 cm^—1^; UV-VIS (MeOH): λ_max_ 363 nm (ε 29,137 cm^−1^M^−1^); HRMS (C_19_H_13_NO) calcd m/z 294.0889 [M+Na]^+^, obsd m/z 294.0887 [M+Na]^+^.

**(*Z*)-4-(2-Fluorophenyl)-5-(3-phenylprop-2-yn-1-ylidene)-1,5-dihydro-2*H*-pyrrol-2-one (25)**

Red semi-solid (75%); ^1^H NMR (CDCl_3_, 400 MHz): δ 5.47 (s, 1H, CH), 6.35 (s, 1H, C3-H), 7.21–7.28 (m, 2H, ArH), 7.38–7.39 (m, 7H, ArH), 7.45–7.53 (m, 4H, ArH), 8.53 (s, 1H, NH); ^13^C NMR (CDCl_3_, 100 MHz): δ 84.8 (C), 92.8 (C), 101.4 (C), 116.3 (ArCH), 118.9 (ArC), 122.4 (CH), 124.2 (ArC), 124.3 (ArCH), 128.0 (ArC), 128.6 (ArCH × 2), 129.1 (ArCH), 130.8 (ArCH), 131.4 (ArCH), 131.8 (ArCH × 2), 143.7 (C), 158.9 (C), 169.5 (C=O); IR (ATR): ν_max_ 745, 831, 1688, 3139 cm^–1^; UV-VIS (MeOH): λ_max_ 364 nm (ε 36,707 cm^−1^M^−1^); HRMS (C_19_H_12_F_1_NO) calcd m/z 312.0795 [M+Na]^+^, obsd m/z 312.0795 [M+Na]^+^.

**(*Z*)-4-(4-Bromophenyl)-5-(3-phenylprop-2-yn-1-ylidene)-1,5-dihydro-2*H*-pyrrol-2-one (26)**

Yellow semi-solid (35%); ^1^H NMR (CDCl_3_, 400 MHz): δ 5.54 (s, 1H, CH), 6.25 (s, 1H, C3-H), 7.31–7.40 (m, 5H, ArH), 7.51–7.54 (m, 2H, ArH), 7.61–7.65 (m, 2H, ArH), 8.09 (s, 1H, NH); ^13^C NMR (CDCl_3_, 100 MHz): δ 84.7 (C), 92.9 (C), 101.7 (C), 121.8 (CH), 122.3 (ArC), 124.3 (ArC), 128.6 (ArCH × 2), 129.2 (ArCH), 130.0 (ArCH × 2), 130.0 (ArC), 131.6 (ArCH), 132.2 (ArCH), 145.5 (C), 149.2 (C), 169.3 (C=O); IR (ATR): ν_max_ 750, 817, 1682, 3138 cm^–1^; UV-VIS (MeOH): λ_max_ 367 nm (ε 34,700 cm^−1^M^−1^); HRMS (C_19_H_12_^79^Br_1_NO) calcd m/z 371.9995 [M+Na]^+^, obsd m/z 371.9995 [M+Na]^+^ and HRMS (C_19_H_12_^81^Br_1_NO) calcd m/z 373.9974 [M+Na]^+^, obsd m/z 373.9974 [M+Na]^+^.

**5-(1,5-Diphenylpenta-1,4-diyn-3-ylidene)-3-ethyl-1-propyl-1,5-dihydro-2*H*-pyrrol-2-one (36)**

Yellow semi-solid (37%); ^1^H NMR (CDCl_3_, 400 MHz): δ 0.93 (t, *J* = 8.0 Hz, 3H, CH_3_), 1.23–1.29 (m, 4H, CH_3_), 1.78–1.83 (m, 2H, CH_2_), 2.44–2.50 (m, 2H, CH_2_), 4.12–4.16 (m, 2H, CH_2_), 7.22 (s, 1H, C4-H), 7.38–7.40 (m, 6H, ArH), 7.52–7.57 (m, 4H, ArH); ^13^C NMR (CDCl_3_, 100 MHz): δ 11.2 (CH_3_), 11.9 (CH_3_), 19.0 (CH_2_), 23.4 (CH_2_), 42.2 (CH_2_), 85.4 (C), 86.2 (C), 92.3 (C), 96.2 (C), 122.6 (C), 128.5 (CH), 128.6 (C), 129.0 (C), 131.4 (C), 131.6 (C), 139.7 (CH), 139.7 (C), 148.4 (C), 170.9 (C=O), IR (ATR): ν_max_ 752, 1040, 1154, 1440, 1569, 1689, 2963 cm^–1^; UV-VIS (MeOH): λ_max_ 395 nm (ε 36,349 cm^−1^M^−1^), 295 (31,086); HRMS (C_26_H_23_NO) calcd m/z 366.1852 [M + H]^+^, obsd m/z 366.1854 [M + H]^+^.

**5-(1,5-Di-*p*-tolylpenta-1,4-diyn-3-ylidene)-3-ethyl-1-propyl-1,5-dihydro-2*H*-pyrrol-2-one (37)**

Yellow semi-solid (38%); ^1^H NMR (CDCl_3_, 400 MHz): δ 0.92 (t, *J* = 8.0 Hz, 3H, CH_3_), 1.22–1.26 (m, 3H, CH_3_), 1.76–1.82 (m, 2H, CH_2_), 2.40–2.43 (bs, 6H, CH_3_), 2.44–2.47 (m, 2H, CH_2_), 4.11–4.15 (m, 2H, CH_2_), 7.18–7.22 (m, 5H, C4-H & ArH), 7.42–7.46 (m, 4H, ArH); ^13^C NMR (CDCl_3_, 100 MHz): δ 11.2 (CH_3_), 12.0 (CH_3_), 19.0 (CH_2_), 21.6 (CH_2_), 23.4 (CH_2_), 42.2 (CH_2_), 85.0 (C), 86.7 (C), 87.0 (C), 92.4 (C), 96.5 (C), 119.6 (C), 129.0 (CH), 129.2 (CH), 131.4 (CH), 138.9 (C), 139.4 (C), 147.9 (C), 170.9 (C=O); IR (ATR): ν_max_ 813, 853, 1197, 1442, 1565, 1694, 2924 cm^–1^; UV-VIS (MeOH): λ_max_ 395 nm (ε 6265 cm^−1^M^−1^), 393 (6186); HRMS (C_28_H_27_NO) calcd m/z 394.2165 [M + H]^+^, obsd m/z 394.2165 [M + H]^+^.

**3-Butyl-5-(1,5-diphenylpenta-1,4-diyn-3-ylidene)-1-propyl-1,5-dihydro-2*H*-pyrrol-2-one (38)**

Yellow semi-solid (48%); ^1^H NMR (CDCl_3_, 400 MHz): δ 0.91–0.99 (m, 6H, CH_3_), 1.40–1.46 (m, 2H, CH_2_), 1.52–1.66 (m, 2H, CH_2_), 1.78–1.83 (m, 2H, CH_2_), 2.42–2.46 (m, 2H, CH_2_), 4.12–4.16 (m, 2H, CH_2_), 7.22 (s, 1H, C4-H), 7.38–7.40 (m, 6H, ArH), 7.52–7.57 (m, 4H, ArH); ^13^C NMR (CDCl_3_, 100 MHz): δ 11.2 (CH_3_), 13.9 (CH_3_), 22.5 (CH_2_), 23.5 (CH_2_), 25.4 (CH_2_), 29.9 (CH_2_), 42.2 (CH_2_), 85.5 (C), 86.2 (C), 86.5 (C), 92.2 (C), 96.2 (C), 122.6 (C), 122.8 (C), 128.4 (CH), 128.5 (CH), 128.7 (C), 129.0 (C), 129.7 (C), 131.4 (CH), 131.5 (CH), 138.3 (C), 148.4 (C), 171.1 (C=O); IR (ATR): ν_max_ 752, 1045, 1322, 1487, 1569, 1689, 2956 cm^–1^; UV-VIS (MeOH): λ_max_ 390 nm (ε 30,999 cm^−1^M^−1^), 300 (26,121); HRMS (C_28_H_27_NO) calcd m/z 394.2165 [M + H]^+^, obsd m/z 394.2164 [M + H]^+^.

**3-Butyl-5-(1,5-di-p-tolylpenta-1,4-diyn-3-ylidene)-1-propyl-1,5-dihydro-2*H*-pyrrol-2-one (39)**

Yellow semi-solid (15%); ^1^H NMR (CDCl_3_, 400 MHz): δ 0.95–0.99 (m, 6H, CH_3_), 1.40–1.45 (m, 2H, CH_2_), 1.60–1.66 (m, 2H, CH_2_), 1.76–1.82 (m, 2H, CH_2_), 2.40 (s, 6H, CH_3_), 2.41–2.47 (m, 2H, CH_2_), 4.11–4.16 (m, 2H, CH_2_), 7.18–24 (m, 5H, ArH & C4-H), 7.33–7.62 (m, 4H, ArH); ^13^C NMR (CDCl_3_, 100 MHz): δ 11.2 (CH_3_), 13.9 (CH_3_), 21.5 (CH_2_), 21.6 (CH_2_), 22.5 (CH_2_), 23.4 (CH_2_), 25.4 (CH_2_), 30.0 (CH_2_), 42.2 (CH_2_), 85.0 (C), 85.7 (C), 86.9 (C), 92.4 (C), 96.5 (C), 119.6 (C), 119.8 (C), 129.2 (CH), 129.2 (CH), 129.5 (C), 129.6 (C), 129.7 (C), 131.3 (CH), 131.5 (CH), 141.4 (C), 147.9 (C), 171.1 (C=O); IR (ATR): ν_max_ 812, 1019, 1170, 1440, 1507, 1689, 2956 cm^–1^; UV-VIS (MeOH): λ_max_ 400 nm (ε 25,717 cm^−1^M^−1^), 300 (25,464); HRMS (C_30_H_31_NO) calcd m/z 422.2478 [M + H]^+^, obsd m/z 422.2475 [M + H]^+^.

**5-(1,5-Diphenylpenta-1,4-diyn-3-ylidene)-3-hexyl-1-propyl-1,5-dihydro-2*H*-pyrrol-2-one (40)**

Yellow semi-solid (39%); ^1^H NMR (CDCl_3_, 400 MHz): δ 0.91–0.98 (m, 6H, CH_3_), 1.32–1.42 (m, 6H, CH_2_), 1.60–1.65 (m, 2H, CH_2_), 1.78–1.83 (m, 2H, CH_2_), 2.41–2.45 (m, 2H, CH_2_), 4.12–4.16 (m, 2H, CH_2_), 7.22 (s, 1H, C4-H), 7.38–7.40 (m, 6H, ArH), 7.52–7.58 (m, 4H, ArH); ^13^C NMR (CDCl_3_, 100 MHz): δ 11.2 (CH_3_), 14.1 (CH_3_), 22.6 (CH_2_), 23.5 (CH_2_), 25.7 (CH_2_), 27.8 (CH_2_), 29.1 (CH_2_), 31.6 (CH_2_), 42.3 (CH_2_), 85.5 (C), 86.2 (C), 86.5 (C), 92.2 (C), 96.2 (C), 122.6 (C), 122.8 (C), 128.4 (CH), 128.5 (CH), 128.7 (C), 129.0 (C), 129.6 (C), 131.4 (CH), 131.6 (CH), 138.4 (C), 148.4 (C), 171.1 (C=O); IR (ATR): ν_max_ 752, 1096, 1154, 1441, 1569, 1690, 2925 cm^–^; UV-VIS (MeOH): λ_max_ 390 nm (ε 34,662 cm^−1^M^−1^), 300 (29,285); HRMS (C_30_H_31_NO) calcd m/z 422.2478 [M + H]^+^, obsd m/z 422.2475 [M + H]^+^.

**5-(1,5-Diphenylpenta-1,4-diyn-3-ylidene)-3-heptyl-1-propyl-1,5-dihydro-2*H*-pyrrol-2-one (41)**

Yellow semi-solid (96%); ^1^H NMR (CDCl_3_, 400 MHz): δ 0.89–0.94 (m, 6H, CH_3_), 1.30–1.34 (m, 8H, CH_2_), 1.36–1.42 (m, 2H, CH_2_), 1.59–1.83 (m, 2H, CH_2_), 2.41–2.45 (m, 2H, CH_2_), 4.12–4.16 (m, 2H, CH_2_), 7.22 (s, 1H, C4-H), 7.38–7.40 (m, 6H, ArH), 7.52–7.57 (m, 4H, ArH); ^13^C NMR (CDCl_3_, 100 MHz): δ 11.2 (CH_3_), 14.1 (CH_3_), 22.7 (CH_2_), 23.4 (CH_2_), 25.7 (CH_2_), 27.9 (CH_2_), 29.0 (CH_2_), 29.4 (CH_2_), 31.7 (CH_2_), 42.2 (CH_2_), 85.5 (C), 86.2 (C), 86.5 (C), 92.2 (C), 96.2 (C), 122.6 (C), 122.8 (C), 128.4 (CH), 128.5 (CH), 128.7 (C), 129.0 (C), 129.6 (C), 131.4 (CH), 131.6 (CH), 138.4 (C), 148.4 (C), 171.1 (C=O); IR (ATR): ν_max_ 752, 1096, 1153, 1322, 1441, 1570, 1693, 2924 cm^–1^; UV-VIS (MeOH): λ_max_ 390 nm (ε 26,340 cm^−1^M^−1^), 295 (24,641); HRMS (C_31_H_33_NO) calcd m/z 436.2635 [M + H]^+^, obsd m/z 436.2633 [M + H]^+^.

**5-(1,5-Diphenylpenta-1,4-diyn-3-ylidene)-3-octyl-1-propyl-1,5-dihydro-2*H*-pyrrol-2-one (42)**

Yellow semi-solid (30%); ^1^H NMR (CDCl_3_, 400 MHz): δ 0.92–0.94 (m, 6H, CH_3_), 1.30–1.40 (m, 10H, CH_2_), 1.60–1.65 (m, 2H, CH_2_), 1.78–1.83 (m, 2H, CH_2_), 2.41–2.45 (m, 2H, CH_2_), 4.12–4.16 (m, 2H, CH_2_), 7.22 (s, 1H, C4-H), 7.38–7.40 (m, 6H, ArH), 7.52–7.57 (m, 4H, ArH); ^13^C NMR (CDCl_3_, 100 MHz): δ 11.2 (CH_3_), 14.1 (CH_3_), 22.7 (CH_2_), 23.5 (CH_2_), 25.7 (CH_2_), 27.9 (CH_2_), 29.2 (CH_2_), 29.3 (CH_2_), 29.4 (CH_2_), 31.8 (CH_2_), 42.2 (CH_2_), 85.5 (C), 86.2 (C), 86.5 (C), 92.2 (C), 96.2 (C), 122.6 (C), 122.8 (C), 128.4 (CH), 128.5 (CH), 128.7 (C), 129.0 (C), 129.6 (C), 131.4 (CH), 131.6 (CH), 138.4 (C), 148.4 (C), 171.1 (C=O); IR (ATR): ν_max_ 752, 1096, 1322, 1440, 1570, 1692, 2923 cm^–1^; UV-VIS (MeOH): λ_max_ 395 nm (ε 47,392 cm^−1^M^−1^), 300 (38,669); HRMS (C_32_H_35_NO) calcd m/z 450.2791 [M + H]^+^, obsd m/z 450.2788 [M + H]^+^.

**1,3-Dibutyl-5-(1,5-diphenylpenta-1,4-diyn-3-ylidene)-1,5-dihydro-2*H*-pyrrol-2-one (43)**

Yellow semi-solid (78%); ^1^H NMR (CDCl_3_, 400 MHz): δ 0.90–0.99 (m, 6H, CH_3_), 1.28–1.48 (m, 4H, CH_2_), 1.56–1.66 (m, 2H, CH_2_), 1.71–1.79 (m, 2H, CH_2_), 2.40–2.46 (m, 2H, CH_2_), 4.15–4.19 (m, 2H, CH_2_), 7.22 (m, 1H, C4-H), 7.36–7.40 (m, 6H, ArH), 7.51–7.58 (m, 4H, ArH); ^13^C NMR (CDCl_3_, 100 MHz): δ 13.8 (CH_3_), 13.9 (CH_3_), 20.0 (CH_2_), 22.5 (CH_2_), 22.4 (CH_2_), 29.9 (CH_2_), 32.4 (CH_2_), 40.6 (CH_2_), 85.5 (C), 86.2 (C), 86.5 (C), 92.2 (C), 96.2 (C), 122.6 (C), 122.8 (C), 128.4 (CH), 128.5 (CH), 128.7 (C), 129.0 (C), 129.7 (C), 131.4 (CH), 131.6 (CH), 138.3 (C), 148.4 (C), 171.1 (C=O); IR (ATR): ν_max_ 751, 801, 1096, 1154, 1437, 1487, 1570, 1689, 2925 cm^−1^; UV-VIS (MeOH): λ_max_ 385 nm (ε 26,773 cm^−1^M^−1^), 300 (25,306); HRMS (C_29_H_29_NO) calcd m/z 408.2322 [M + H]^+^, obsd m/z 408.2323 [M + H]^+^.

**3-Butyl-5-(penta-1,4-diyn-3-ylidene)-1-propyl-1,5-dihydro-2*H*-pyrrol-2-one (44)**

Yellow semi-solid (31%); ^1^H NMR (CDCl_3_, 400 MHz): δ 0.86–0.95 (m, 6H, CH_3_), 1.35–1.40 (m, 2H, CH_2_), 1.52–1.68 (m, 4H, CH_2_), 2.35–2.39 (m, 2H, CH_2_), 3.21 (s, 1H), 3.41 (s, 1H), 3.93–3.97 (m, 2H, CH_2_), 7.07 (s, 1H, C4-H); ^13^C NMR (CDCl_3_, 100 MHz): δ 10.6 (CH_3_), 13.8 (CH_3_), 22.4 (CH_2_), 23.3 (CH_2_), 25.2 (CH_2_), 29.7 (CH_2_), 41.9 (CH_2_), 79.0 (C), 79.9 (C), 80.4 (C), 83.2 (C), 84.4 (C), 129.6 (CH), 139.2 (C), 151.2 (C), 170.9 (C=O); IR (ATR): ν_max_ 696, 855, 1018, 1169, 1364, 1377, 1457, 1694, 2930, 2959 cm^−1^; UV-VIS (MeOH): λ_max_ 305 nm (ε 2558 cm^−1^M^−1^); HRMS (C_16_H_19_NO) calcd m/z 242.1539 [M + H]^+^, obsd m/z 242.1540 [M + H]^+^.

## 4. Conclusions

Fifteen novel alkyne analogues of DHPs with various substitution patterns and aliphatic chain lengths were successfully synthesized via lactamisation and Sonogashira coupling reactions in moderate to high yields. The Sonogashira reaction was carried out with DHPs and alkynes in the presence of CuI, palladium catalyst PdCl_2_(PPh_3_)_2,_ and TEA. Biological testing demonstrated that several compounds showed moderate activity against the *P. aeruginosa* MH602 reporter strain with little bactericidal effect. The present study represents the first application of the Sonogashira reaction to DHP scaffolds for the synthesis of novel bacterial QS inhibitors.

## Figures and Tables

**Table 1:** Percentage QS inhibition of the tested compounds against *P. aeruginosa* reporter strain MH602.

Compound	Concentration (µM)		
	250	125	62.5
**16**	^b^ 58.6 ± 2.1	^a^ 47.5 ± 6.5	^a^ 44.7 ± 4.6
**17**	^b^ 46.4 ± 1.1	^a^ 32.9 ± 1.4	^a^ 19.5 ± 1.9
**18**	^a^ 52.2 ± 8.7	^a^ 51.0 ± 1.8	^a^ 47.8 ± 0.8
**19**	^a^ 43.9 ± 2.9	^a^ 25.0 ± 2.7	^a^ 24.1 ± 9.3
**20**	^a^ 59.2 ± 0.4	^a^ 44.8 ± 3.4	^a^ 39.3 ± 3.6
**24**	^a^ 49.2 ± 3.8	^a^ 35.2 ± 4.5	^a^ 28.5 ± 0.7
**25**	^a^ 46.6 ± 4.8	^a^ 30.7 ± 4.5	^a^ 20.0 ± 7.2
**26**	^a^ 54.8 ± 5.6	^a^ 39.7 ± 1.5	^a^ 30.2 ± 6.7
**36**	^b^ 70.0 ± 3.6	^a^ 55.2 ± 1.1	^a^ 36.4 ± 8.3
**37**	^a^ 45.4 ± 1.3	^a^ 28.0 ± 1.5	^a^ 25.1 ± 2.8
**38**	^a^ 67.6 ± 0.6	^a^ 57.8 ± 5.9	^a^ 40.6 ± 3.3
**39**	^b^ 65.8 ± 4.6	^a^ 63.0 ± 4.5	^a^ 54.1 ± 4.4
**40**	^b^ 71.2 ± 8.1	^a^ 65.3 ± 1.6	^a^ 52.9 ± 6.7
**41**	^b^ 68.6 ± 3.1	^a^ 53.3 ± 8.1	^a^ 43.3 ± 6.1
**42**	^a^ 66.1 ± 6.4	^a^ 54.8 ± 6.2	^a^ 39.9 ± 8.9
**43**	^a^ 59.5 ± 0.3	^a^ 48.7 ± 3.2	^a^ 24.3 ± 1.9
**44**	^b^ 79.8 ± 0.8	^a^ 66.1 ± 6.2	^a^ 55.8 ± 4.6
**^d^** **1**	^c^ 84.8 ± 2.9	^c^ 82.5 ± 2.5	^c^ 74.4 ± 3.6

^a^ Bacterial growth inhibition ≤ 15%; ^b^ growth inhibition between 15 and 30%; ^c^ growth inhibition greater than 30%; ^d^ positive control; ± the standard deviation of the mean for at least three independent experiments. In each independent experiment, compounds were tested in triplicate.

## Data Availability

Data is contained within the article and Supplementary Materials.

## Supplementary Materials

The following are available online at https://www.mdpi.com/article/10.3390/antibiotics11020151/s1: Table S1: X-ray Experimental details for compound **18**, Table S2: X-ray Experimental details for compound **26**, Table S3: Selected geometric parameters, Table S4: Selected hydrogen-bond parameters, File S1: NMR spectra for ^1^H NMR and ^13^C NMR.
