# Supplementary material for: Synthesis of Alkyne-Substituted Dihydropyrrolones as Bacterial Quorum-Sensing Inhibitors of Pseudomonas aeruginosa
Source: Antibiotics (Basel). 2022 Jan 25;11(2):151. doi: 10.3390/antibiotics11020151 (PMC8868272; doi:10.3390/antibiotics11020151)

Supplementary Materials

# Synthesis of Alkyne-Substituted Dihydropyrrolones as Bacterial Quorum Sensing Inhibitors of *Pseudomonas aeruginosa*

**Basmah Almohaywi [1], Tsz Tin Yu [2,*], George Iskander [2], Shekh Sabir [2], Mohan Bhadbhade [3], David StC. Black [2], and Naresh Kumar [2,*]**

1 Department of Pharmaceutical Chemistry, College of Pharmacy, King Khalid University Abha 6142, Saudi Arabia; bal-mohawe@kku.edu.sa

2 School of Chemistry, The University of New South Wales, Sydney, NSW 2052, Australia; g.iskanderb@gmail.com (G.I.); s.sabir@student.unsw.edu.au (S.S.); d.black@unsw.edu.au (D.StC.B.)

3 Solid State and Elemental Analysis Unit, Mark Wainwright Analytical Centre, Division of Research, The University of New South Wales, Sydney, NSW 2052, Australia; m.bhadbhade@unsw.edu.au

* Correspondence: tsztin.yu@unsw.edu.au (T.T.Y.); n.kumar@unsw.edu.au (N.K.)

## Table of Contents

Crystal Structure Details .......... 2
NMR Spectral Data .......... 6
3-Butyl-5-(1,5-diphenylpenta-1,4-diyn-3-ylidene)-1,5-dihydro-2*H*-pyrrol-2-one (18) .......... 6
3-butyl-5-(1,5-di-p-tolylpenta-1,4-diyn-3-ylidene)-1,5-dihydro-2*H*-pyrrol-2-one (19) .......... 7
5-(1,5-Diphenylpenta-1,4-diyn-3-ylidene)-3-hexyl-1,5-dihydro-2*H*-pyrrol-2-one (20) .......... 8
(*Z*)-4-Phenyl-5-(3-phenylprop-2-yn-1-ylidene)-1,5-dihydro-2*H*-pyrrol-2-one (24) .......... 9
(*Z*)-4-(2-Fluorophenyl)-5-(3-phenylprop-2-yn-1-ylidene)-1,5-dihydro-2*H*-pyrrol-2- one (25) .......... 10
(*Z*)-4-(4-Bromophenyl)-5-(3-phenylprop-2-yn-1-ylidene)-1,5-dihydro-2*H*-pyrrol-2- one (26) .......... 11
5-(1,5-Diphenylpenta-1,4-diyn-3-ylidene)-3-ethyl-1-propyl-1,5-dihydro-2*H*-pyrrol- 2-one (36) .......... 12
5-(1,5-Di-*p*-tolylpenta-1,4-diyn-3-ylidene)-3-ethyl-1-propyl-1,5-dihydro-2*H*-pyrrol- 2-one (37) .......... 13
3-Butyl-5-(1,5-diphenylpenta-1,4-diyn-3-ylidene)-1-propyl-1,5-dihydro-2*H*-pyrrol- 2-one (38) .......... 14
3-Butyl-5-(1,5-di-p-tolylpenta-1,4-diyn-3-ylidene)-1-propyl-1,5-dihydro-2*H*-pyrrol- 2-one (39) .......... 15
5-(1,5-Diphenylpenta-1,4-diyn-3-ylidene)-3-hexyl-1-propyl-1,5-dihydro-2*H*-pyrrol- 2-one (40) .......... 16
5-(1,5-Diphenylpenta-1,4-diyn-3-ylidene)-3-heptyl-1-propyl-1,5-dihydro-2*H*- pyrrol-2-one (41) .......... 17
5-(1,5-Diphenylpenta-1,4-diyn-3-ylidene)-3-octyl-1-propyl-1,5-dihydro-2*H*-pyrrol- 2-one (42) .......... 18
1,3-Dibutyl-5-(1,5-diphenylpenta-1,4-diyn-3-ylidene)-1,5-dihydro-2*H*-pyrrol-2-one (43) .......... 19
3-Butyl-5-(penta-1,4-diyn-3-ylidene)-1-propyl-1,5-dihydro-2*H*-pyrrol-2-one (44) .......... 20

## Crystal Structure Details

**Table S1. Experimental details for compound 18**

| | |
|---|---|
| Crystal data | |
| Chemical formula | $C_{25}H_{21}NO$ |
| $M_r$ | 351.43 |
| Crystal system, space group | Monoclinic, $P2_1/c$ |
| Temperature (K) | 150 |
| $a$, $b$, $c$ (Å) | 8.8068 (7), 20.8376 (15), 10.6832 (6) |
| β (°) | 104.884 (2) |
| $V$ (Å$^3$) | 1894.7 (2) |
| $Z$ | 4 |
| Radiation type | Mo *K*α |
| μ (mm$^{-1}$) | 0.07 |
| Crystal size (mm) | 0.05 × 0 × 0 |
| Data collection | |
| Diffractometer | Bruker D8 Quest |
| Absorption correction | Multi-scan<br>*SADABS2016*/2 (Bruker,2016/2) was used for absorption correction. wR2(int) was 0.1421 before and 0.0847 after correction. The Ratio of minimum to maximum transmission is 0.7998. The λ/2 correction factor is Not present. |
| $T_{min}$, $T_{max}$ | 0.596, 0.746 |
| No. of measured, independent and observed [$I > 2\sigma(I)$] reflections | 52956, 3532, 2377 |
| $R_{int}$ | 0.119 |
| (sin θ/λ)$_{max}$ (Å$^{-1}$) | 0.606 |
| Refinement | |
| $R[F^2 > 2\sigma(F^2)]$, $wR(F^2)$, $S$ | 0.049, 0.146, 1.12 |
| No. of reflections | 3532 |
| No. of parameters | 245 |
| H-atom treatment | H-atom parameters constrained |
| $\Delta\rho_{max}$, $\Delta\rho_{min}$ (e Å$^{-3}$) | 0.19, −0.19 |

Computer programs: *SAINT* V8.38A (Bruker, 2018), *SHELXT* 2014/5 (Sheldrick, 2014), *SHELXL* 2018/3 (Sheldrick, 2015), Olex2 1.5 (Dolomanov *et al.*, 2009).

**Table S2. X-ray Experimental details for compound 26**

| | |
|---|---|
| Chemical formula | $C_{19}H_{12}BrNO$ |
| $M_r$ | 350.21 |
| Crystal system, space group | Monoclinic, $P2_1/c$ |
| Temperature (K) | 105 |
| *a*, *b*, *c* (Å) | 11.944 (4), 9.578 (3), 13.798 (4) |
| β (°) | 105.385 (6) |
| $V$ (Å$^3$) | 1521.9 (8) |
| $Z$ | 4 |
| Radiation type | Mo *K*α |
| μ (mm$^{-1}$) | 2.70 |
| Crystal size (mm) | 0.27 × 0.09 × 0.03 |
| Data collection | |
| Diffractometer | Bruker D8Quest |
| Absorption correction | Multi-scan<br>*SADABS2016*/2 (Bruker,2016/2) was used for absorption correction. wR2(int) was 0.1015 before and 0.0523 after correction. The Ratio of minimum to maximum transmission is 0.8415. The λ/2 correction factor is Not present. |
| $T_{min}$, $T_{max}$ | 0.627, 0.746 |
| No. of measured, independent and observed [$I > 2\sigma(I)$] reflections | 47699, 3512, 2917 |
| $R_{int}$ | 0.045 |
| $(\sin \theta/\lambda)_{max}$ (Å$^{-1}$) | 0.651 |
| Refinement | |
| $R[F^2 > 2\sigma(F^2)]$, $wR(F^2)$, $S$ | 0.026, 0.068, 1.05 |
| No. of reflections | 3512 |
| No. of parameters | 199 |
| H-atom treatment | H-atom parameters constrained |
| $\Delta\rho_{max}$, $\Delta\rho_{min}$ (e Å$^{-3}$) | 0.34, −0.51 |

Computer programs: *SAINT* V8.38A, *SHELXT* 2014/5, *SHELXL*, Olex2

**Table S3. Selected geometric parameters (Å, º)**

| | | | |
|---|---|---|---|
| Br01—C8 | 1.9009 (19) | C7—C8 | 1.381 (3) |
| C1—C2 | 1.345 (3) | C8—C9 | 1.381 (3) |
| C1—C4 | 1.470 (3) | C9—C10 | 1.384 (3) |
| C1—C5 | 1.476 (2) | C11—C12 | 1.420 (3) |
| N1—C3 | 1.370 (2) | C12—C13 | 1.199 (3) |
| N1—C4 | 1.390 (2) | C13—C14 | 1.436 (3) |
| O1—C3 | 1.234 (2) | C14—C15 | 1.396 (3) |
| C2—C3 | 1.470 (3) | C14—C19 | 1.397 (3) |
| C4—C11 | 1.346 (3) | C15—C16 | 1.384 (3) |
| C5—C6 | 1.397 (3) | C16—C17 | 1.383 (3) |
| C5—C10 | 1.394 (3) | C17—C18 | 1.379 (3) |
| C6—C7 | 1.389 (3) | C18—C19 | 1.390 (3) |
| | | | |
| C2—C1—C4 | 107.83 (16) | C7—C8—Br01 | 120.05 (14) |
| C2—C1—C5 | 127.53 (17) | C9—C8—Br01 | 118.16 (14) |
| C4—C1—C5 | 124.41 (16) | C9—C8—C7 | 121.76 (17) |
| C3—N1—C4 | 110.91 (15) | C8—C9—C10 | 118.86 (18) |
| C1—C2—C3 | 108.96 (17) | C9—C10—C5 | 120.77 (18) |
| N1—C3—C2 | 106.07 (16) | C4—C11—C12 | 122.31 (17) |
| O1—C3—N1 | 125.35 (17) | C13—C12—C11 | 176.4 (2) |
| O1—C3—C2 | 128.59 (18) | C12—C13—C14 | 179.2 (2) |
| N1—C4—C1 | 106.13 (16) | C15—C14—C13 | 119.68 (18) |
| C11—C4—C1 | 128.18 (17) | C15—C14—C19 | 119.65 (18) |
| C11—C4—N1 | 125.31 (16) | C19—C14—C13 | 120.66 (18) |
| C6—C5—C1 | 122.30 (17) | C16—C15—C14 | 119.87 (19) |
| C10—C5—C1 | 118.44 (17) | C17—C16—C15 | 120.29 (19) |
| C10—C5—C6 | 119.26 (17) | C18—C17—C16 | 120.20 (19) |
| C7—C6—C5 | 120.20 (17) | C17—C18—C19 | 120.3 (2) |
| C8—C7—C6 | 119.14 (18) | C18—C19—C14 | 119.64 (19) |

**Table S4. Selected hydrogen-bond parameters**

| *D*—H···*A* | *D*—H (Å) | H···*A* (Å) | *D*···*A* (Å) | *D*—H···*A* (°) |
|---|---|---|---|---|
| N1—H1···O1$^{i}$ | 0.88 | 2.01 | 2.855 (2) | 161.0 |
| C15—H15···O1$^{i}$ | 0.95 | 2.67 | 3.341 (3) | 127.9 |

Symmetry code(s): (i) -*x*+1, -*y*+1, -*z*+2.

## Supplementary File S1

## NMR Spectral Data

## 3-Butyl-5-(1,5-diphenylpenta-1,4-diyn-3-ylidene)-1,5-dihydro-2*H*-pyrrol-2-one (18)

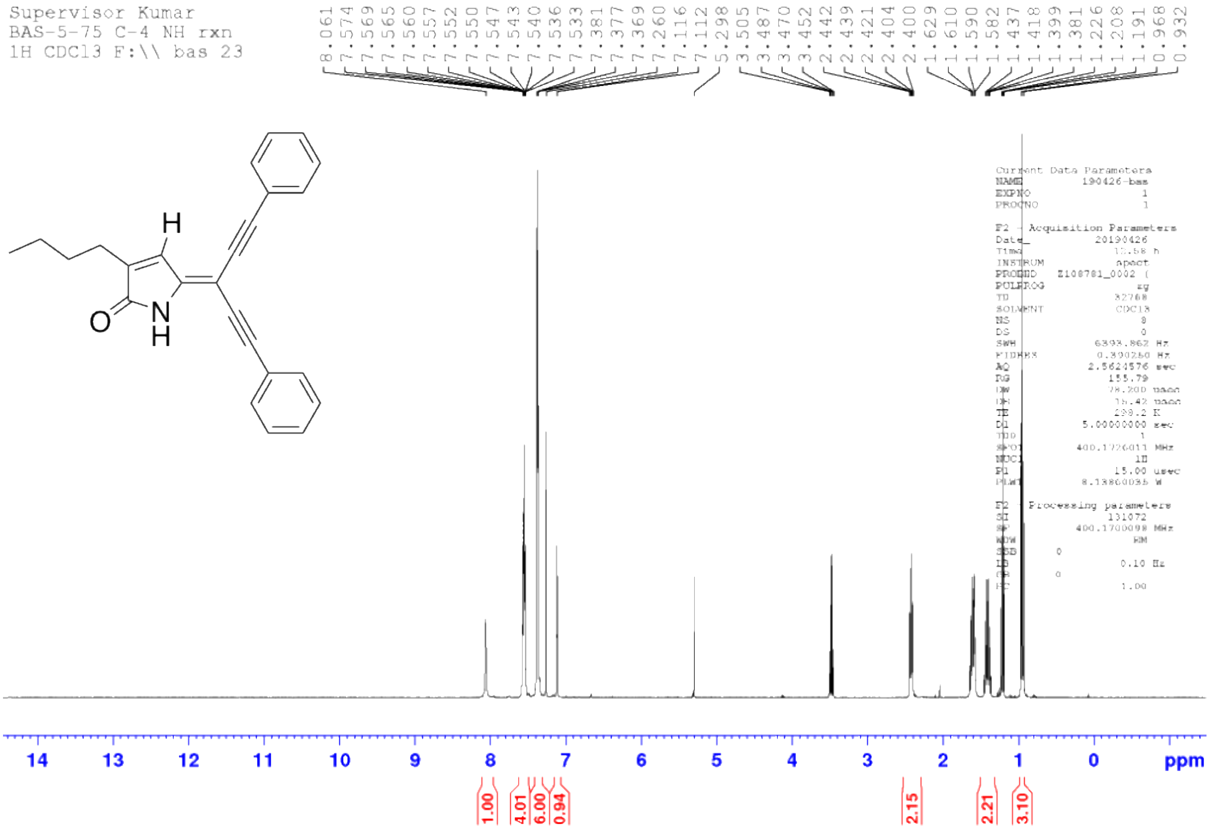


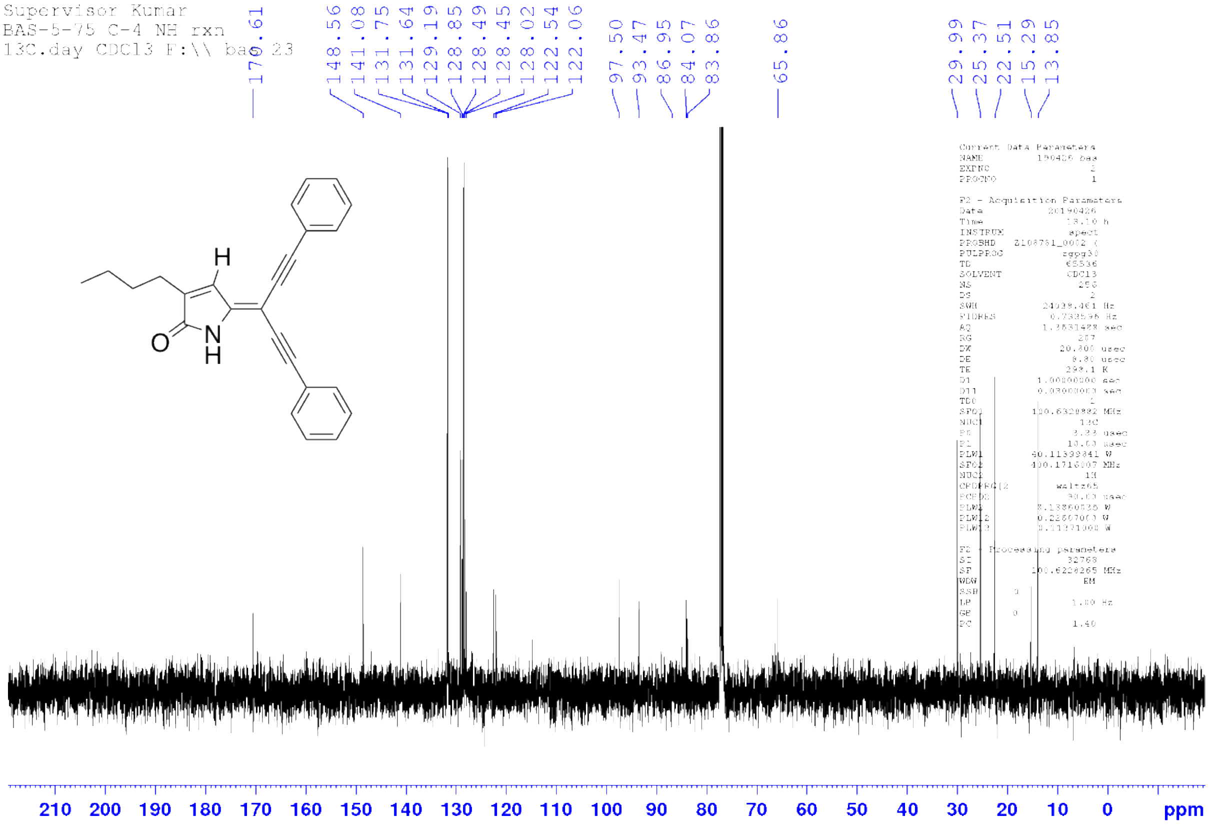

## 3-butyl-5-(1,5-di-p-tolylpenta-1,4-diyn-3-ylidene)-1,5-dihydro-2*H*-pyrrol-2-one (19)

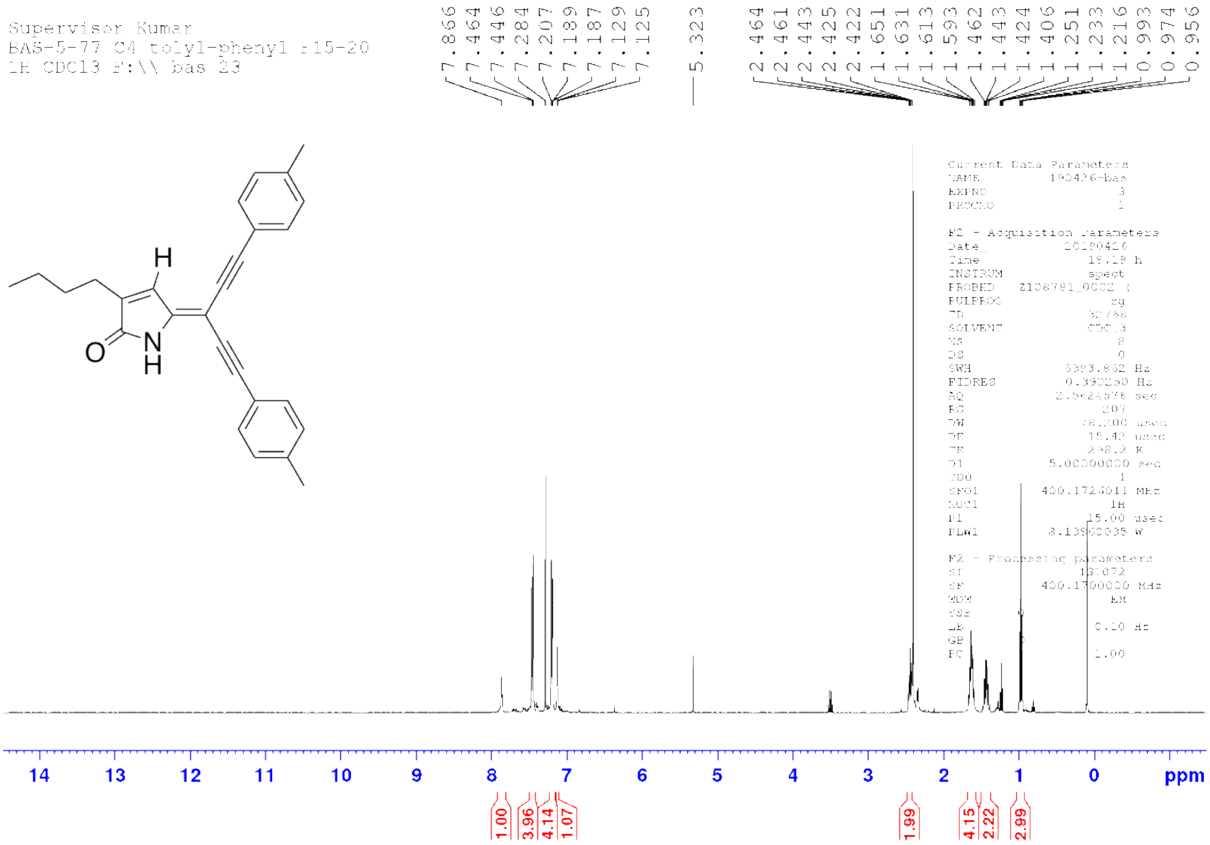

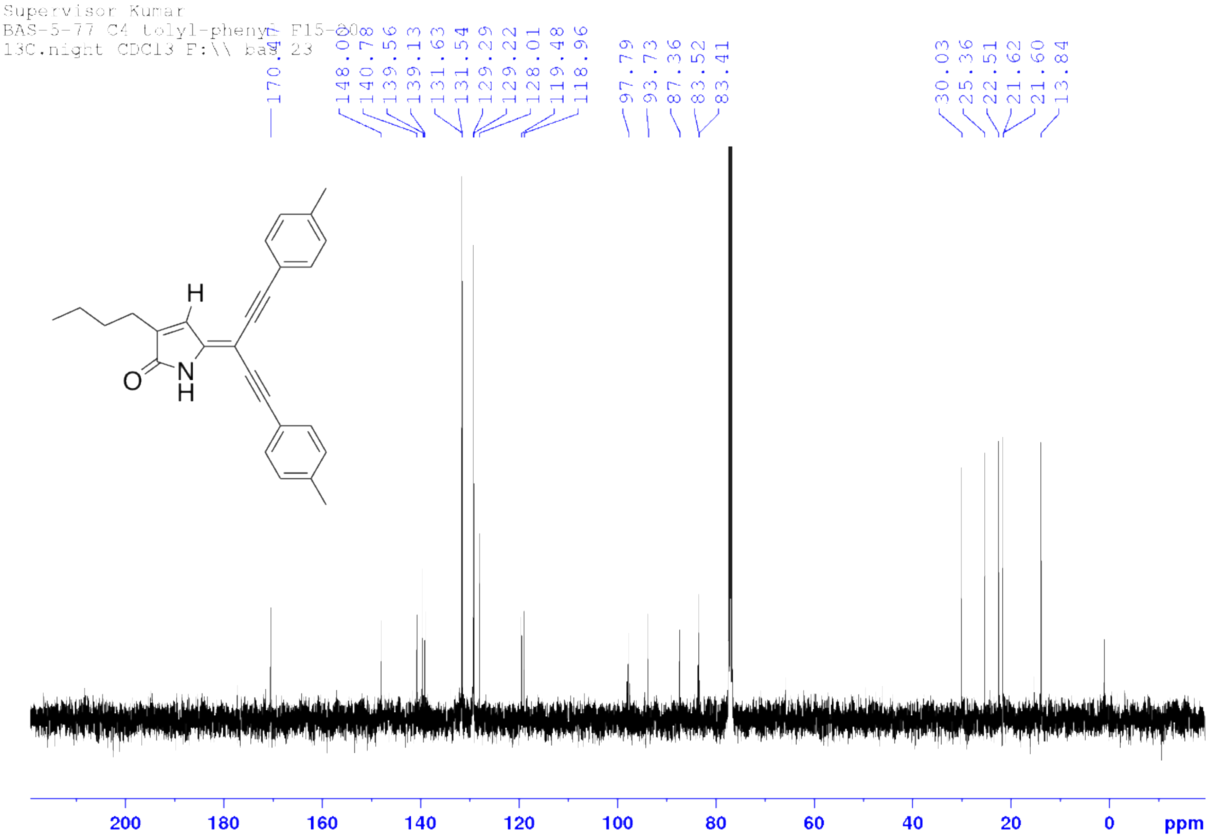

# 5-(1,5-Diphenylpenta-1,4-diyn-3-ylidene)-3-hexyl-1,5-dihydro-2*H*-pyrrol-2-one (20)

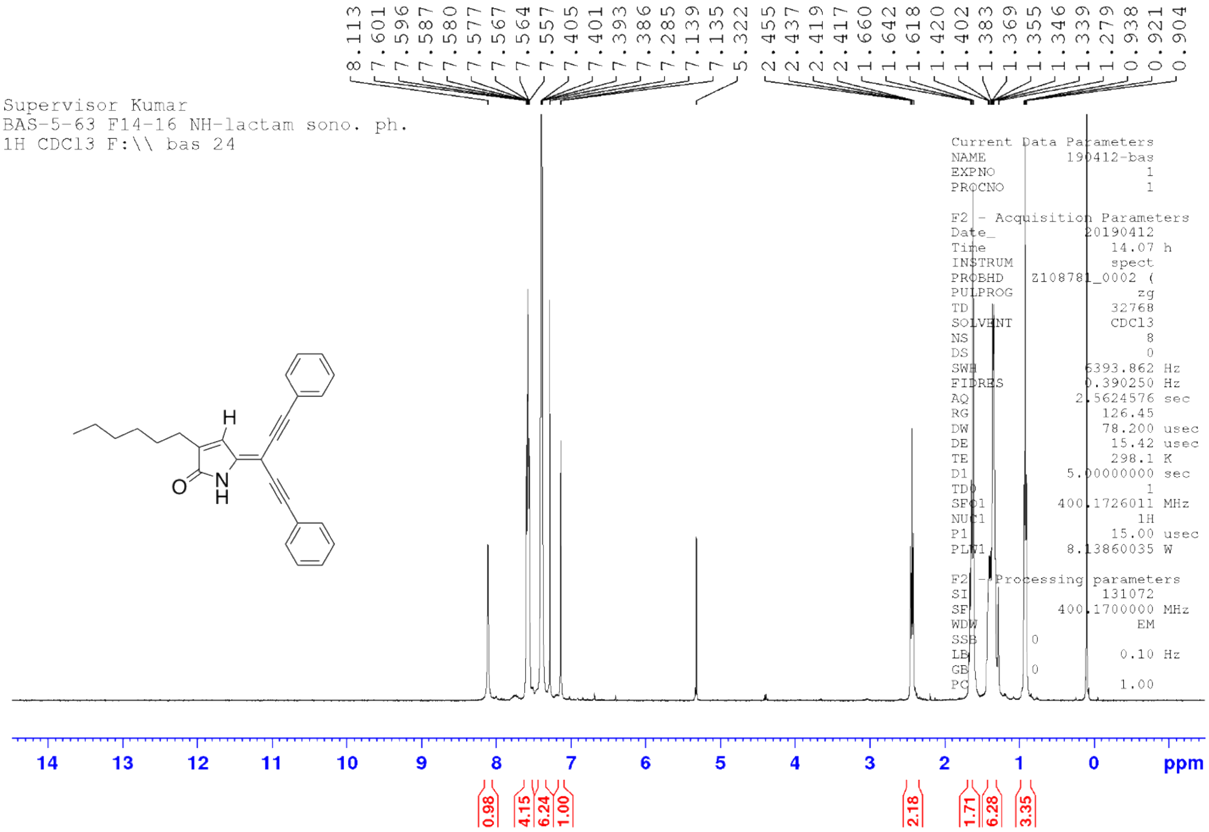


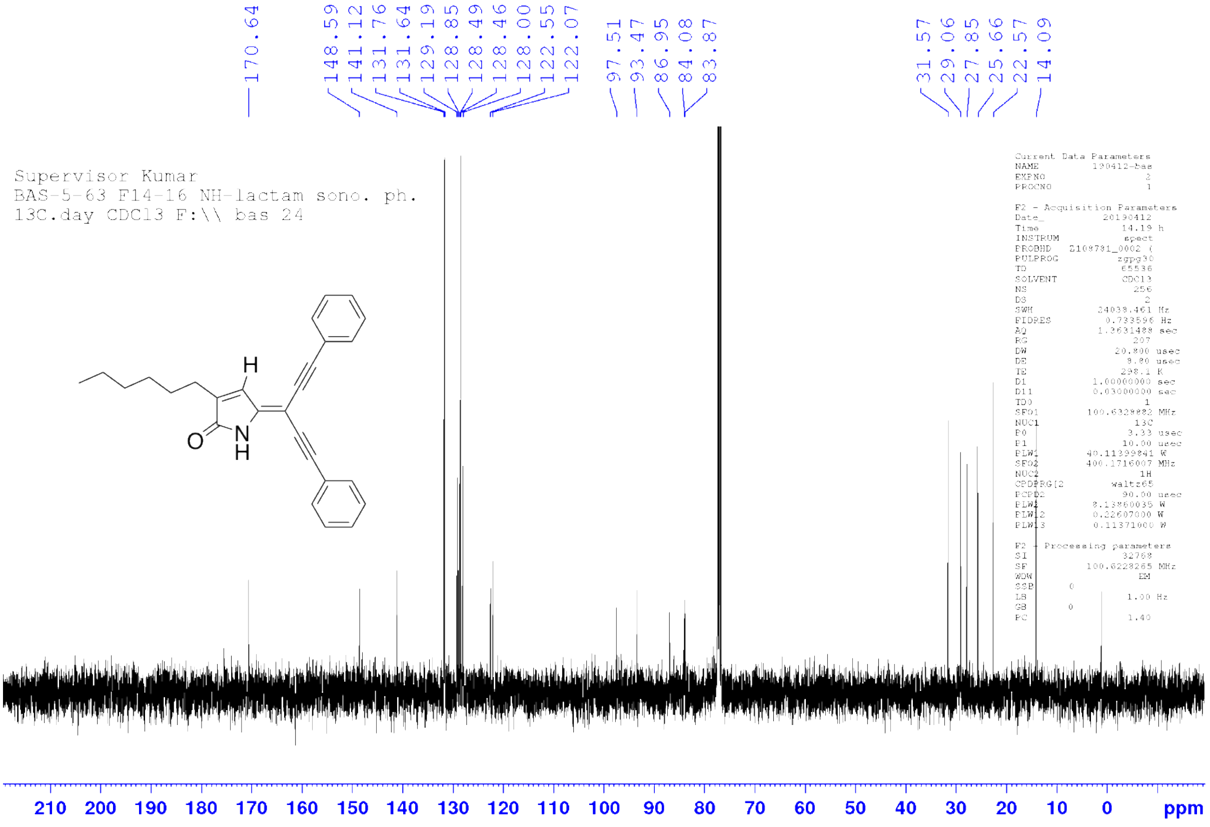

## (*Z*)-4-Phenyl-5-(3-phenylprop-2-yn-1-ylidene)-1,5-dihydro-2*H*-pyrrol-2-one (24)

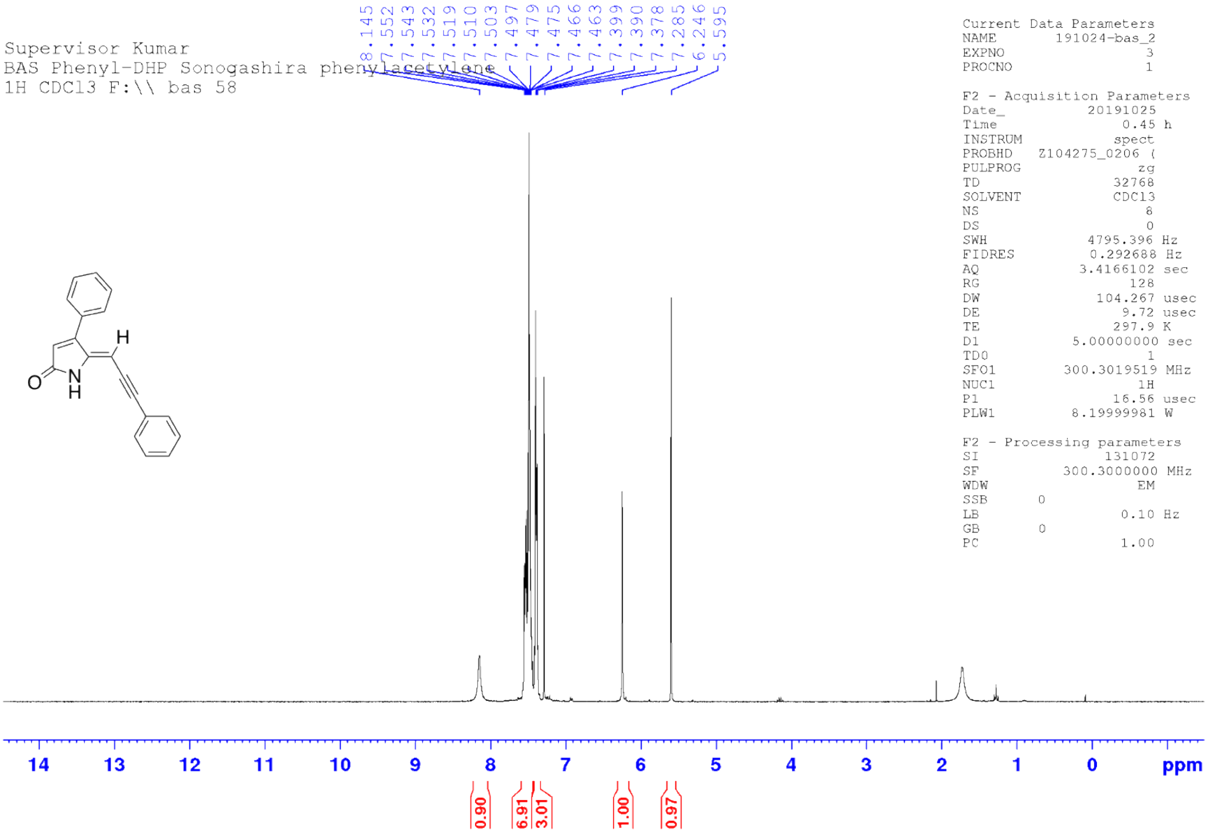


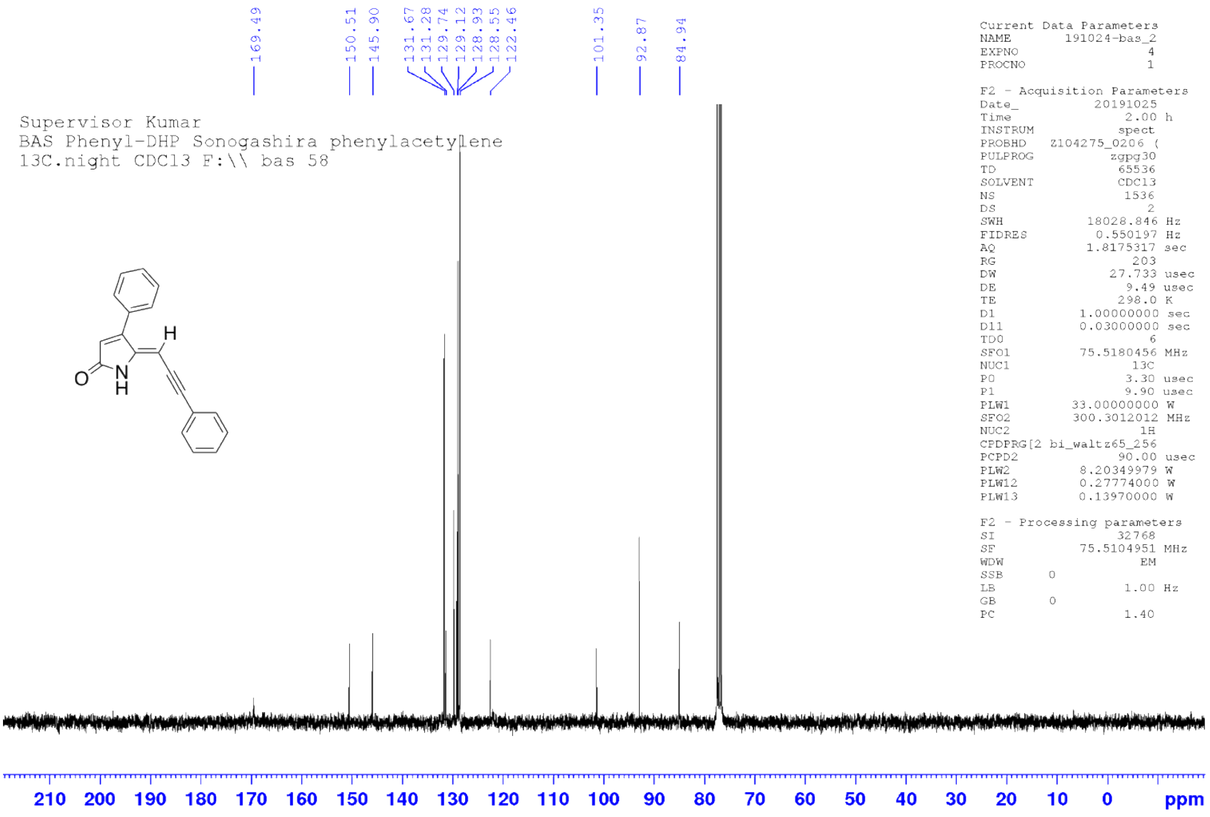

## (*Z*)-4-(2-Fluorophenyl)-5-(3-phenylprop-2-yn-1-ylidene)-1,5-dihydro-2*H*-pyrrol-2- one (25)

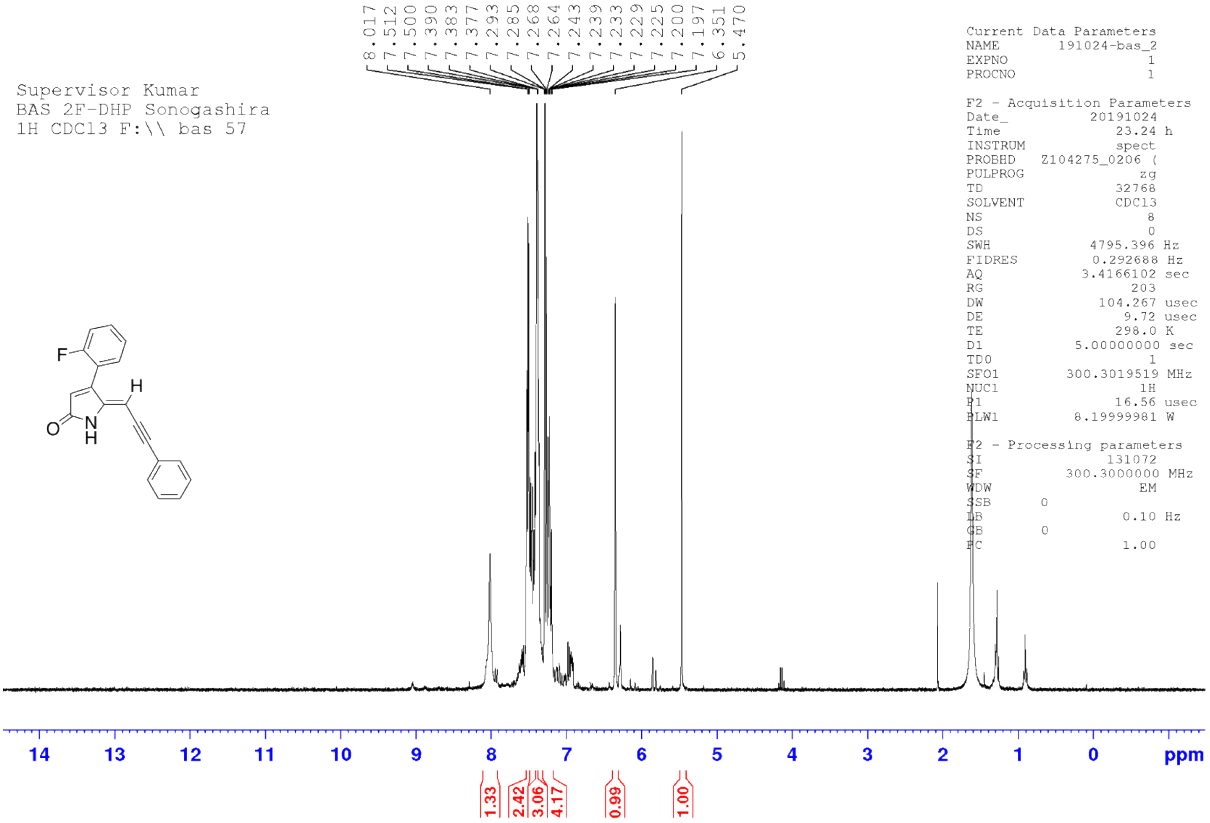


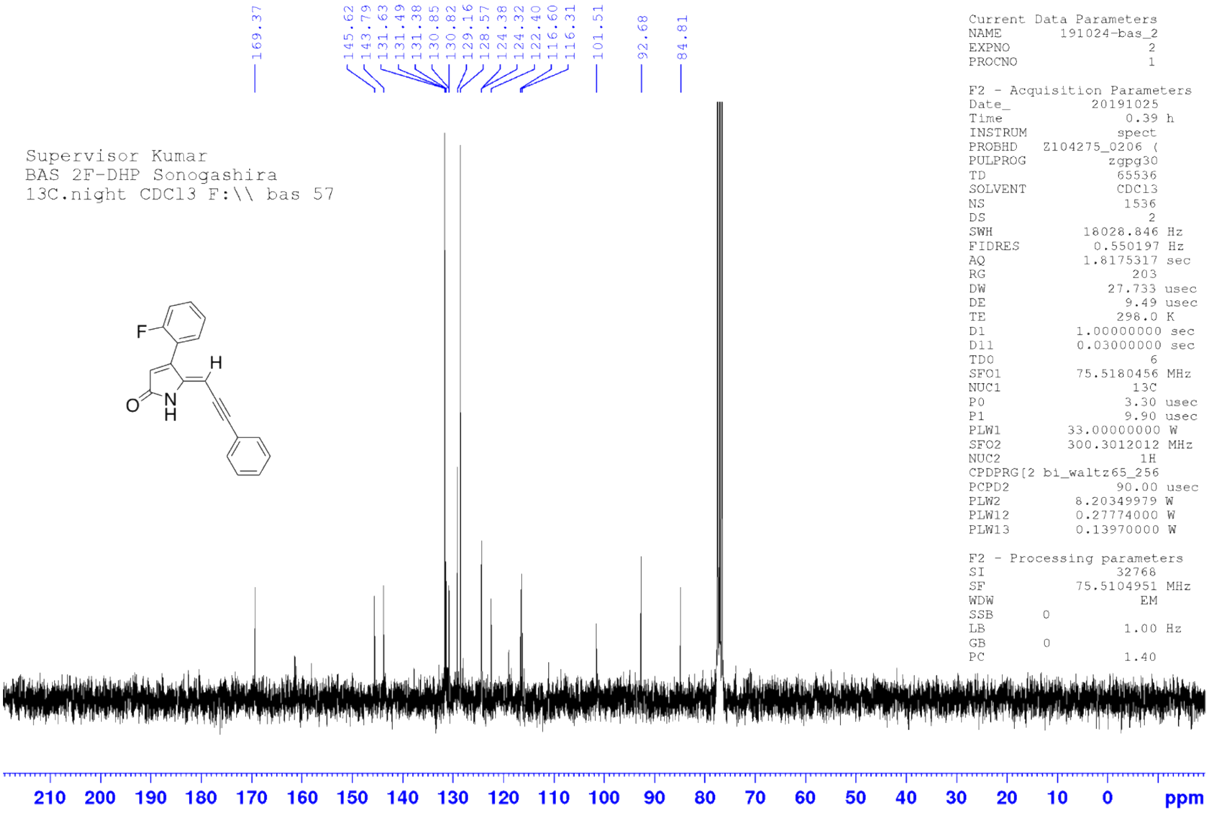

## (*Z*)-4-(4-Bromophenyl)-5-(3-phenylprop-2-yn-1-ylidene)-1,5-dihydro-2*H*-pyrrol-2- one (26)

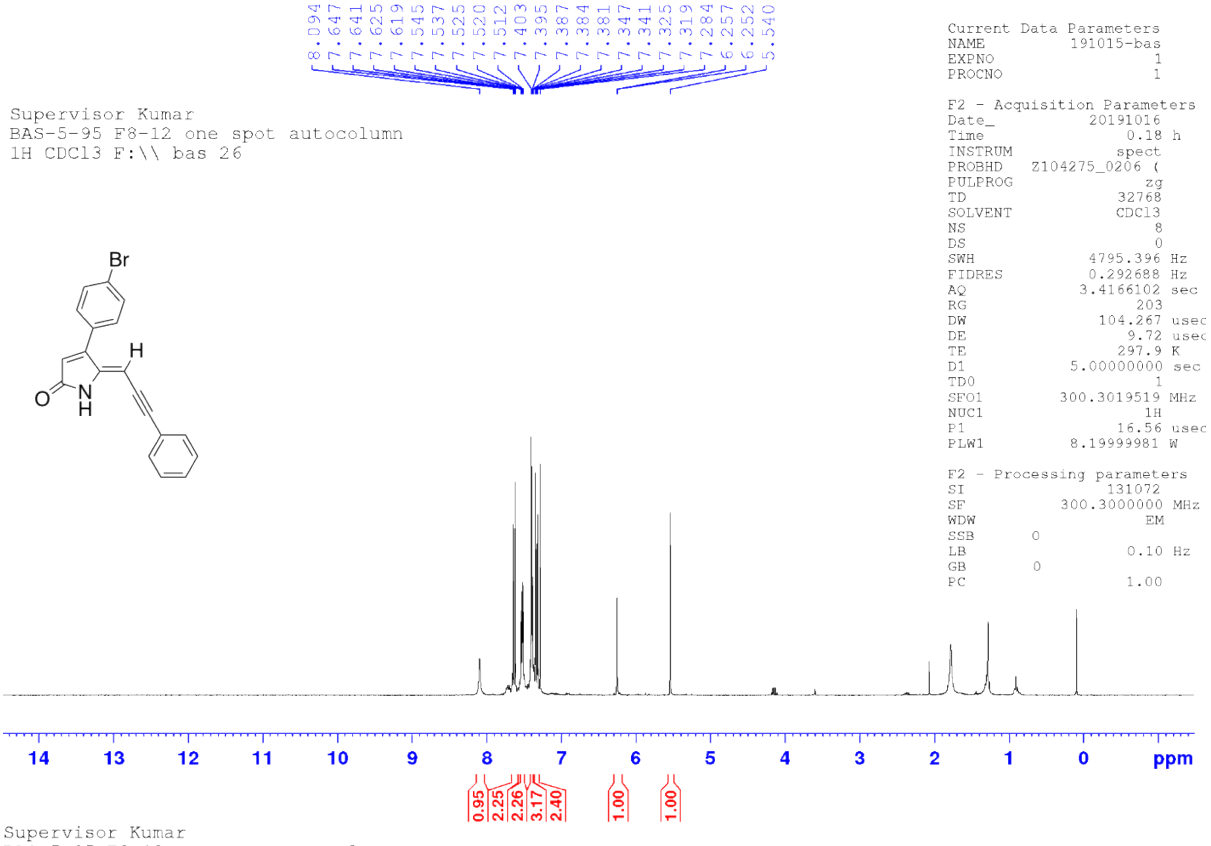


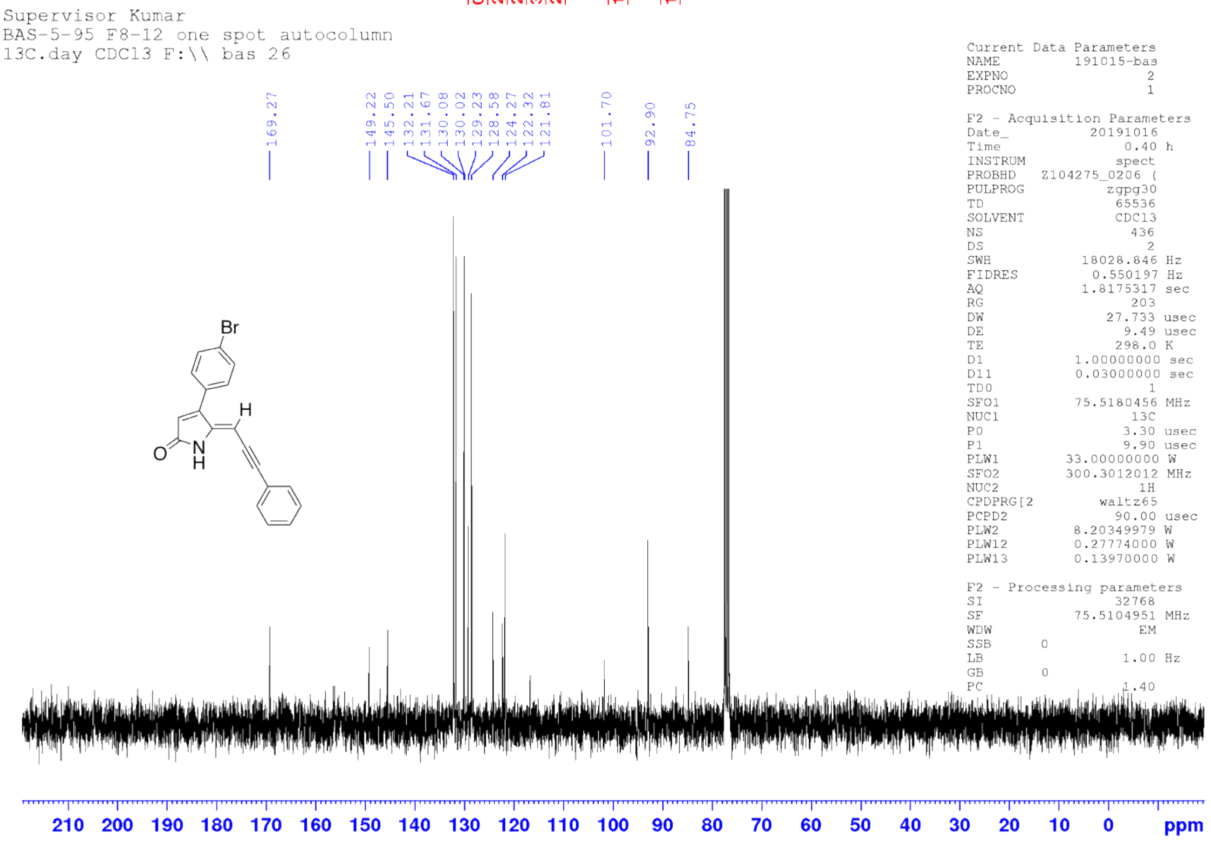

## 5-(1,5-Diphenylpenta-1,4-diyn-3-ylidene)-3-ethyl-1-propyl-1,5-dihydro-2*H*-pyrrol- 2-one (36)

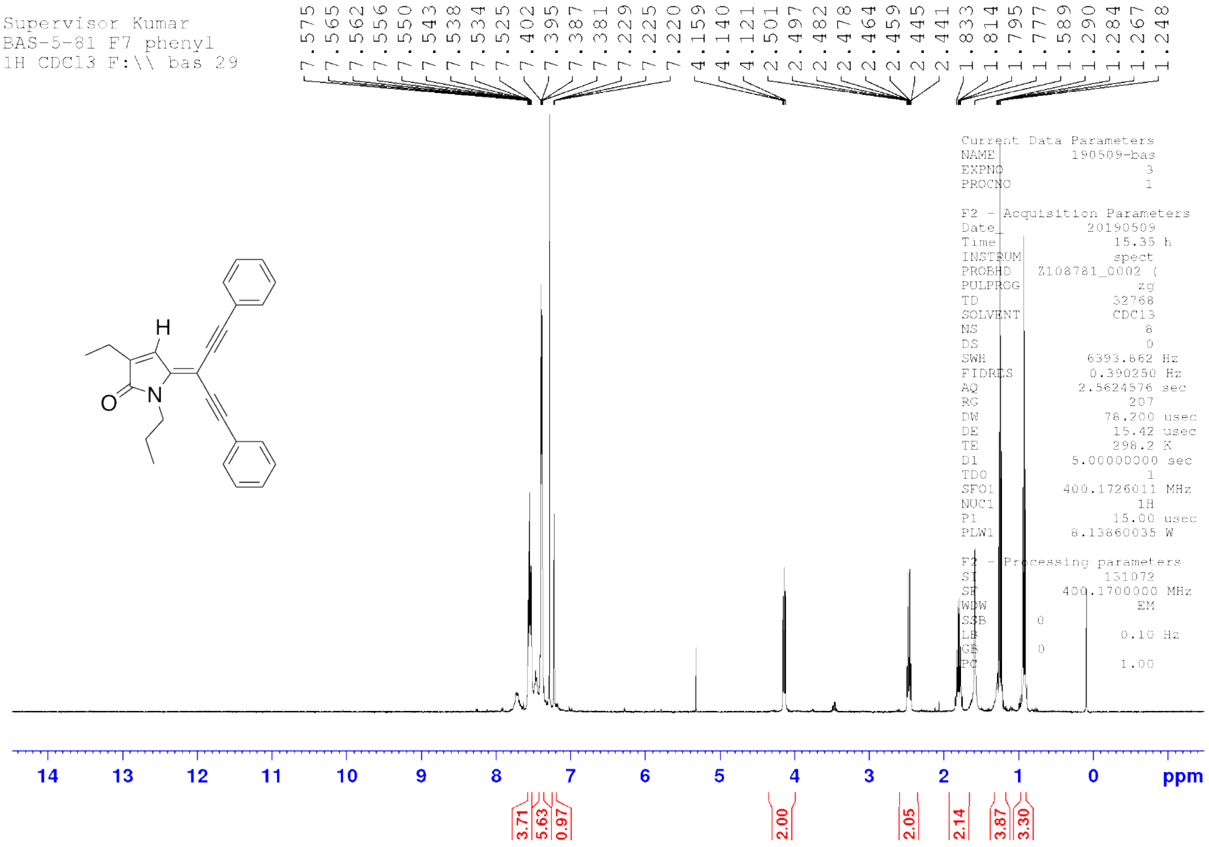


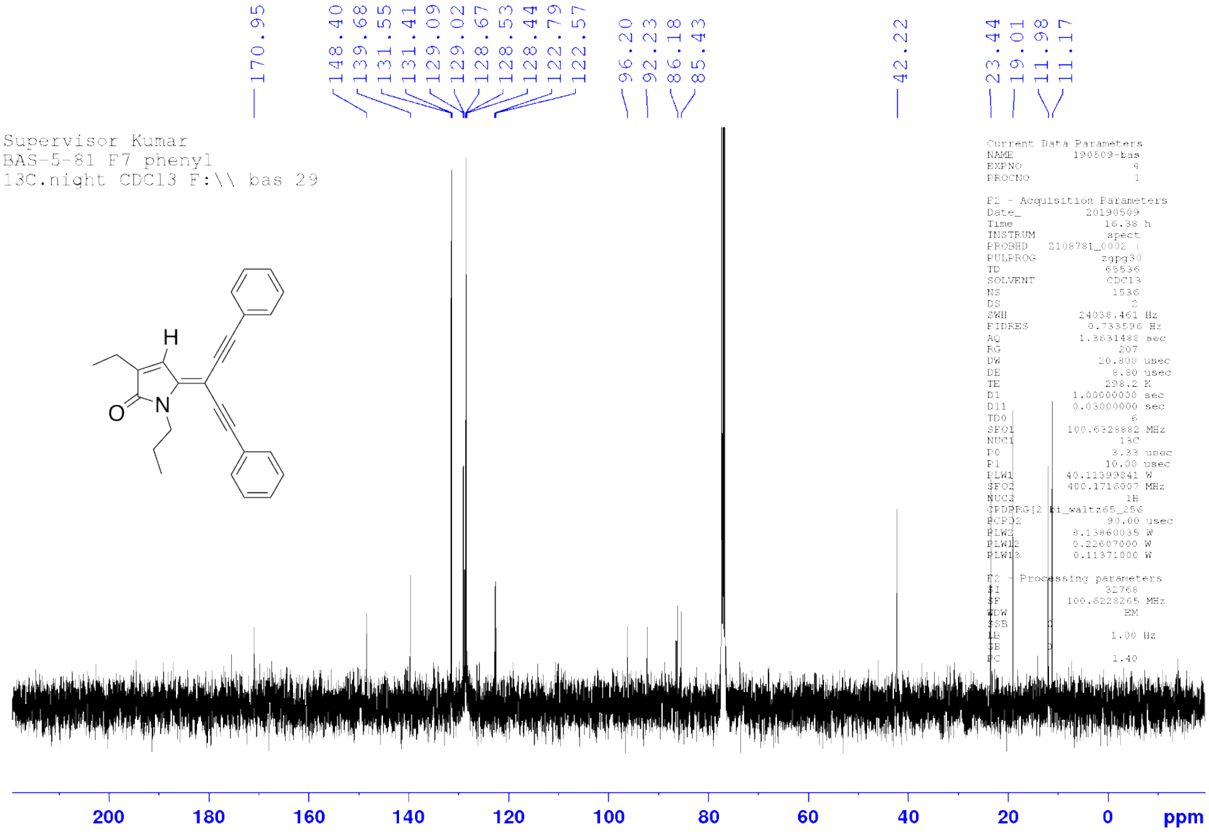

5-(1,5-Di-*p*-tolylpenta-1,4-diyn-3-ylidene)-3-ethyl-1-propyl-1,5-dihydro-2*H*-pyrrol- 2-one (37)

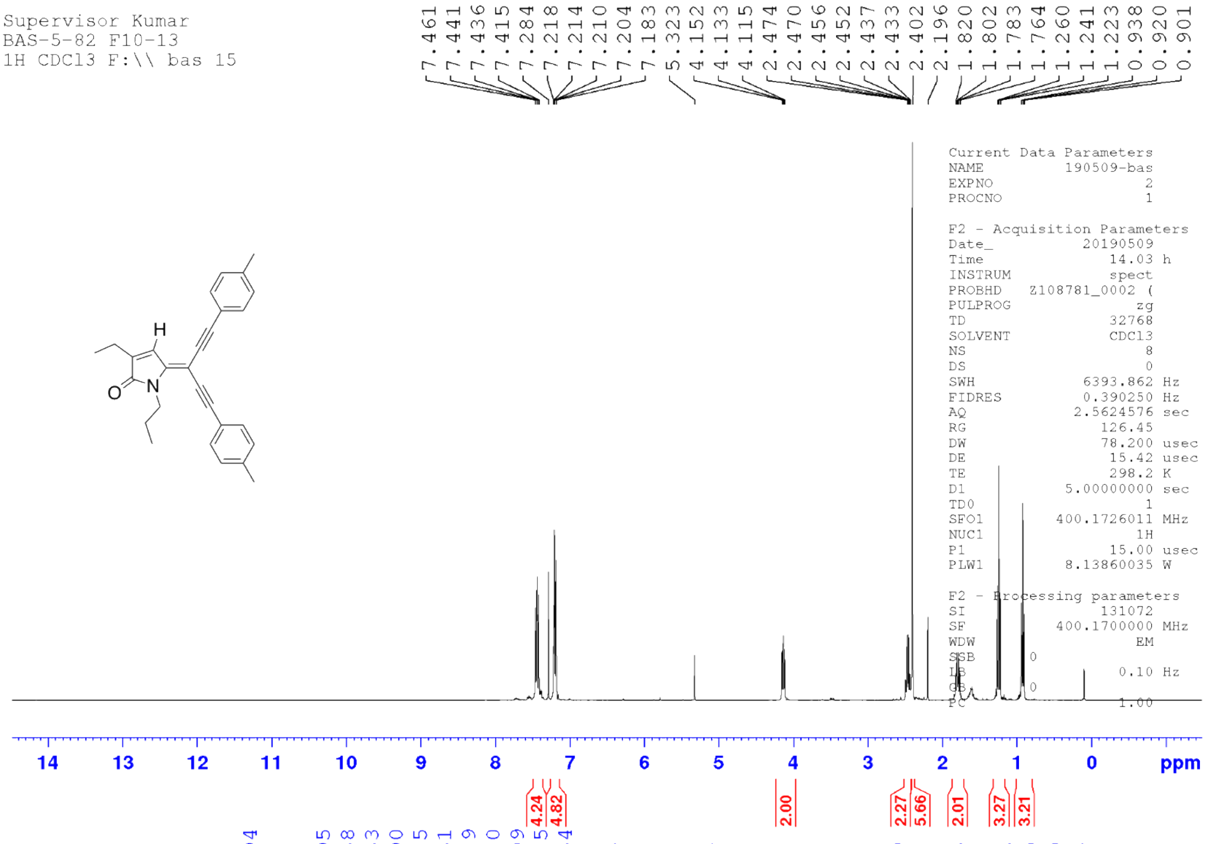


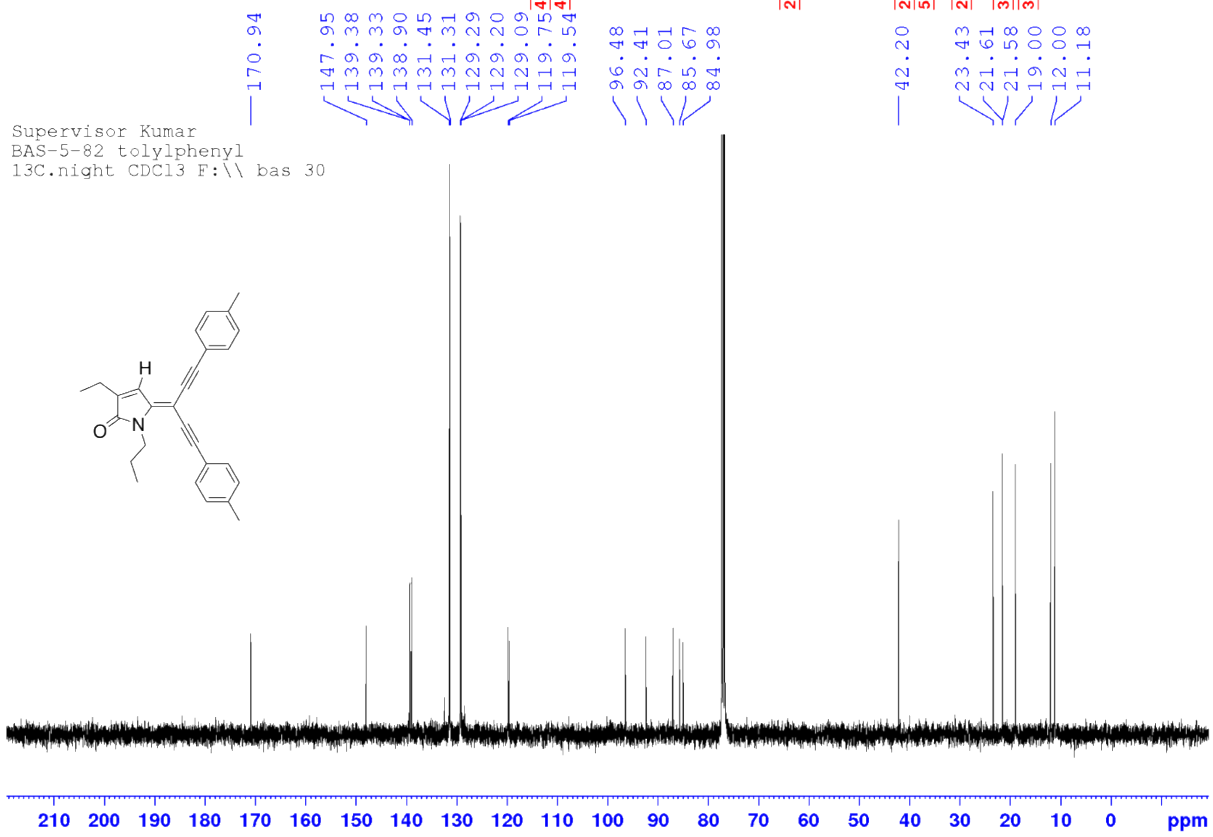

# 3-Butyl-5-(1,5-diphenylpenta-1,4-diyn-3-ylidene)-1-propyl-1,5-dihydro-2*H*-pyrrol- 2-one (38)

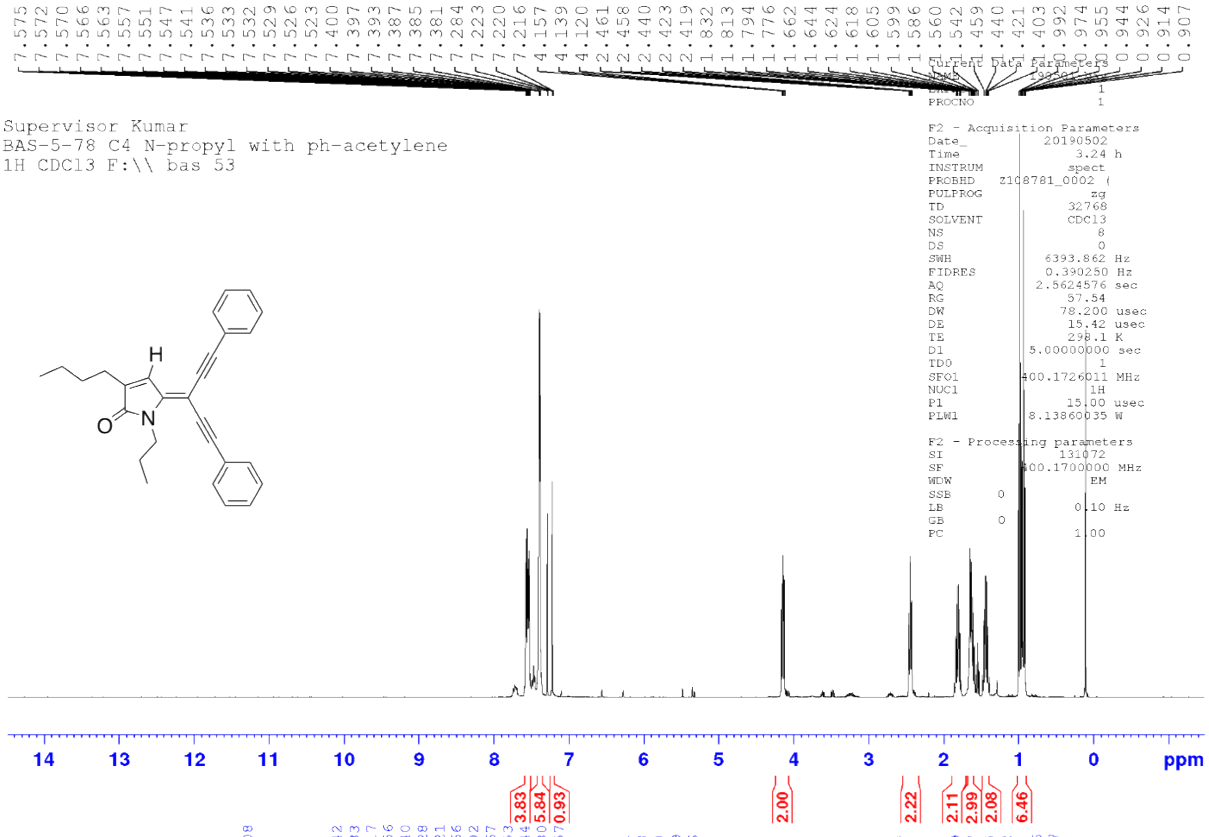


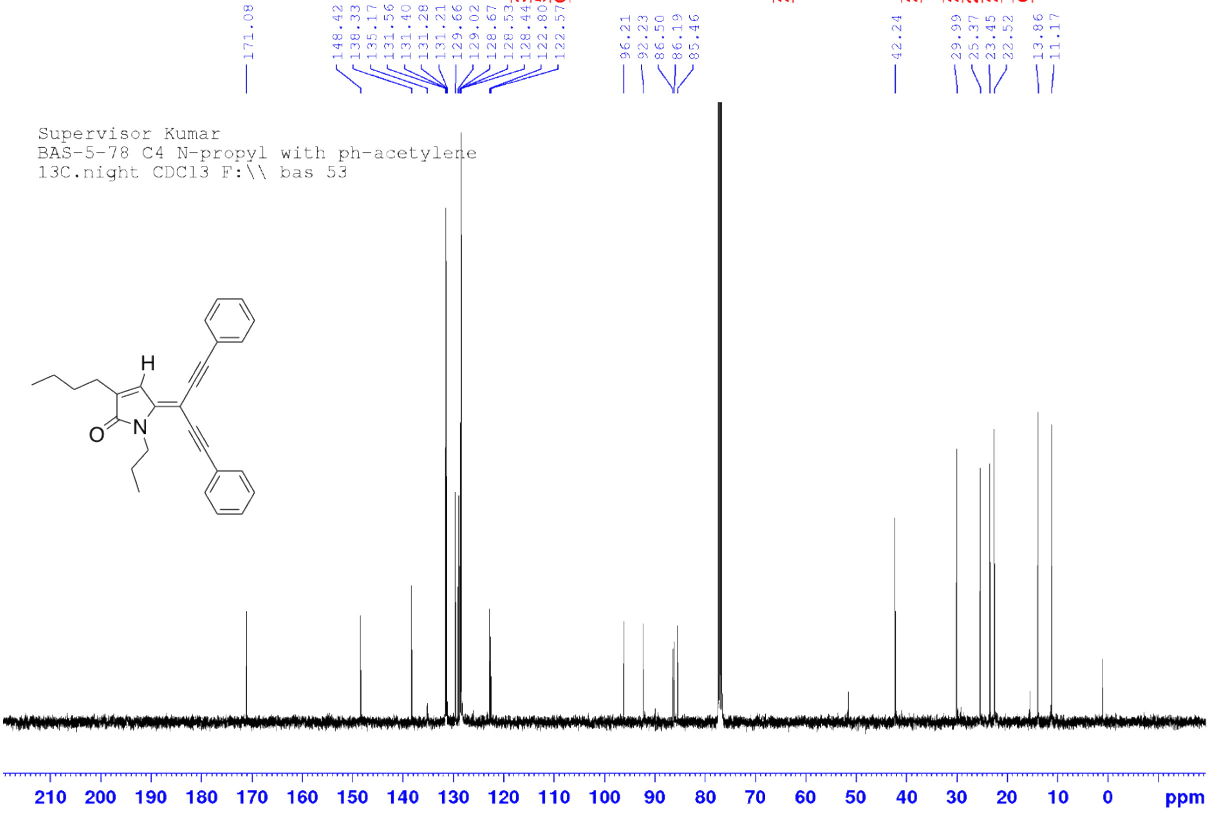

## 3-Butyl-5-(1,5-di-p-tolylpenta-1,4-diyn-3-ylidene)-1-propyl-1,5-dihydro-2*H*-pyrrol- 2-one (39)

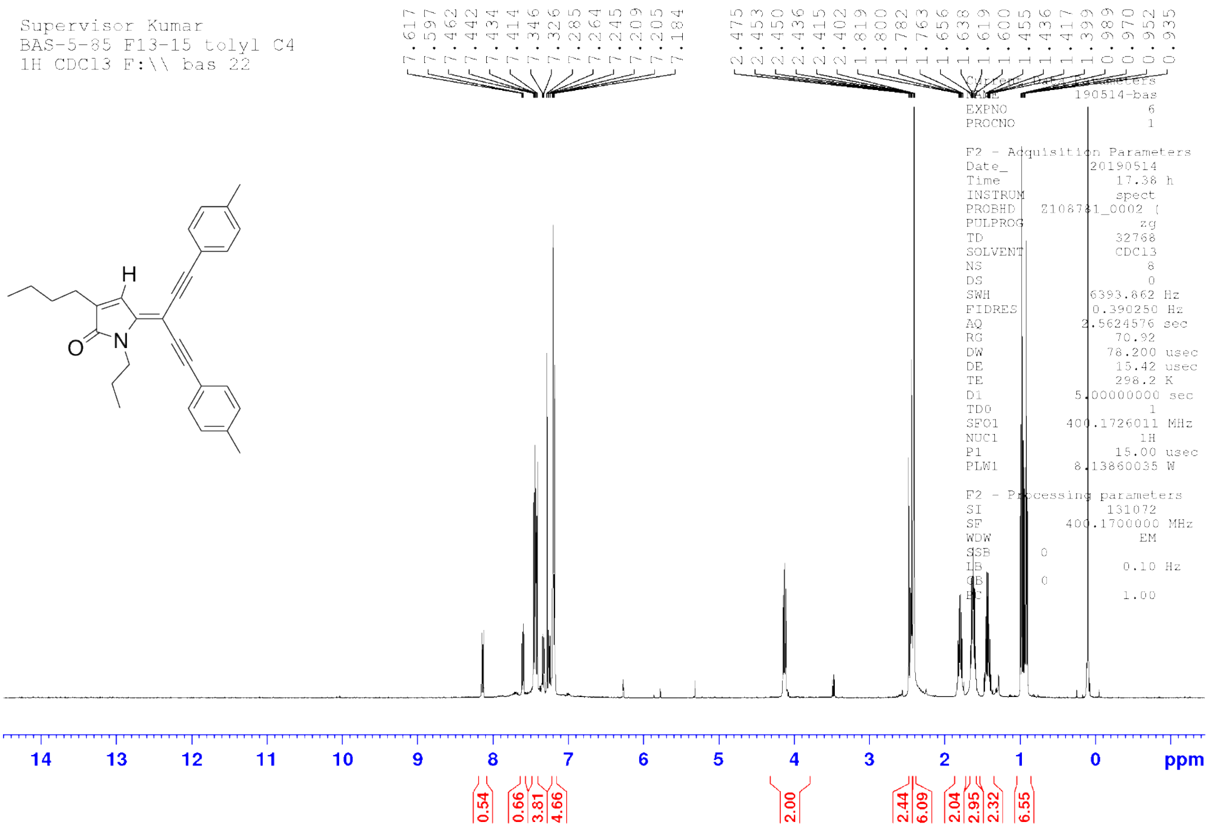


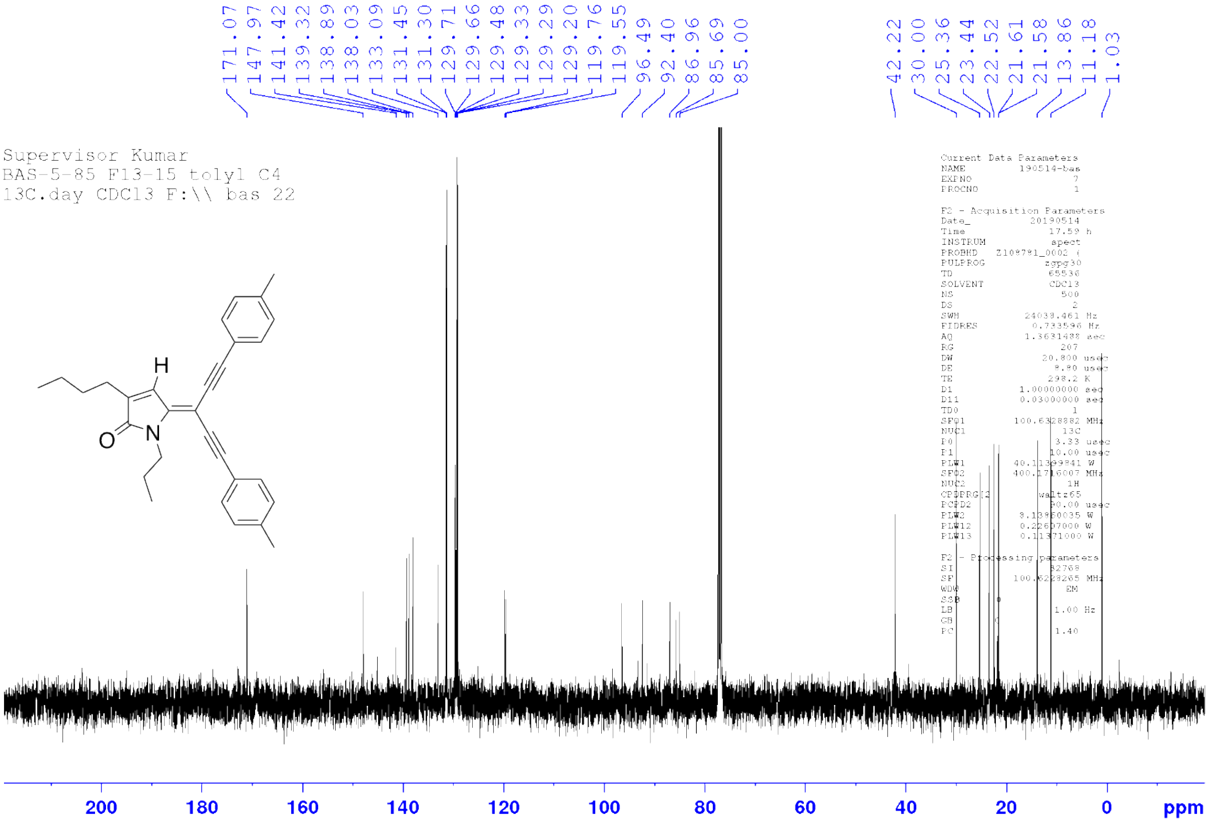

5-(1,5-Diphenylpenta-1,4-diyn-3-ylidene)-3-hexyl-1-propyl-1,5-dihydro-2*H*-pyrrol- 2-one (40)

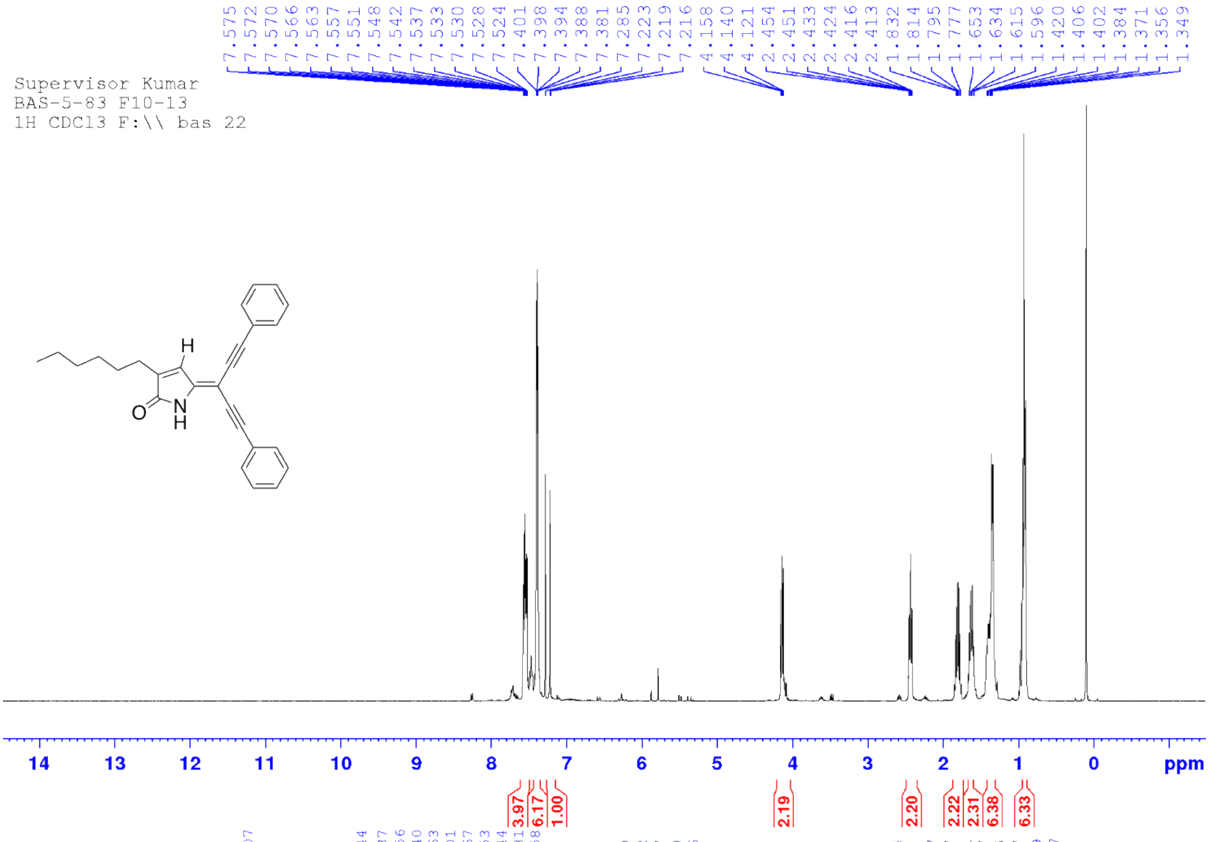

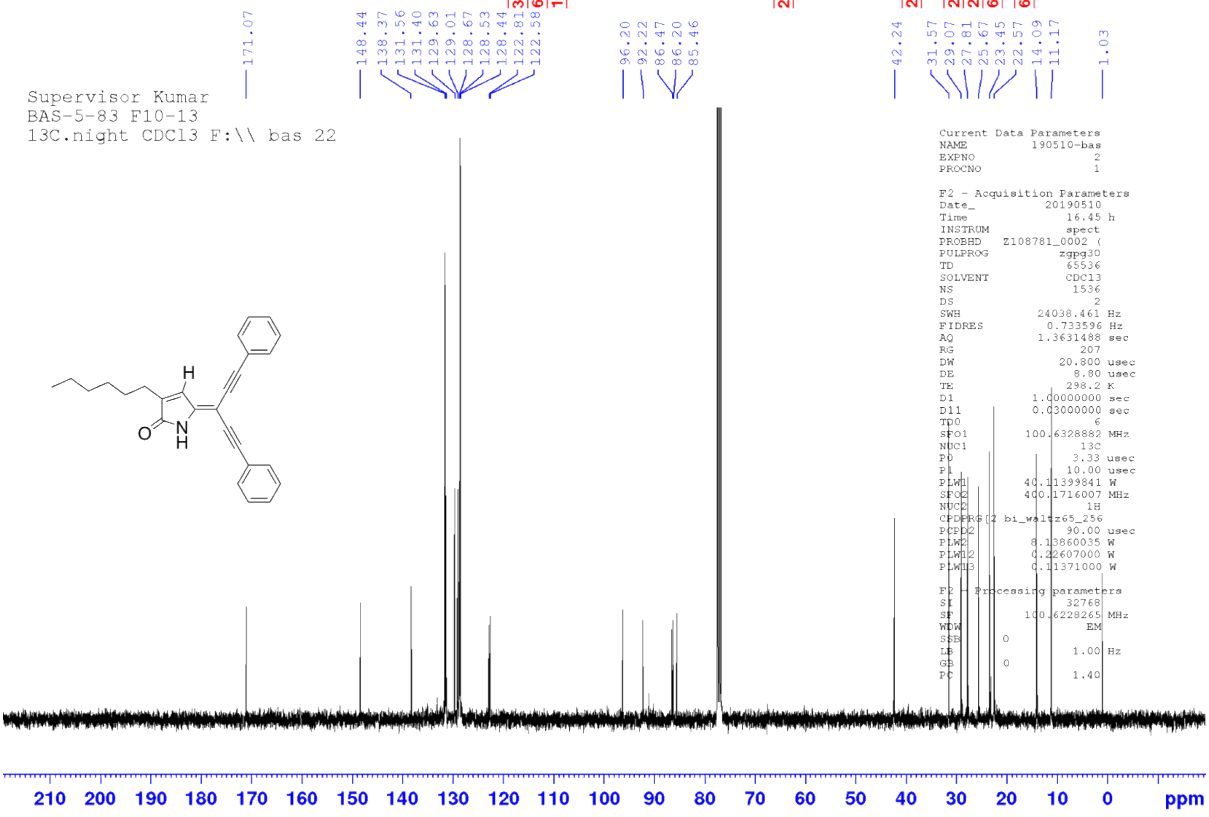

5-(1,5-Diphenylpenta-1,4-diyn-3-ylidene)-3-heptyl-1-propyl-1,5-dihydro-2*H*- pyrrol-2-one (41)

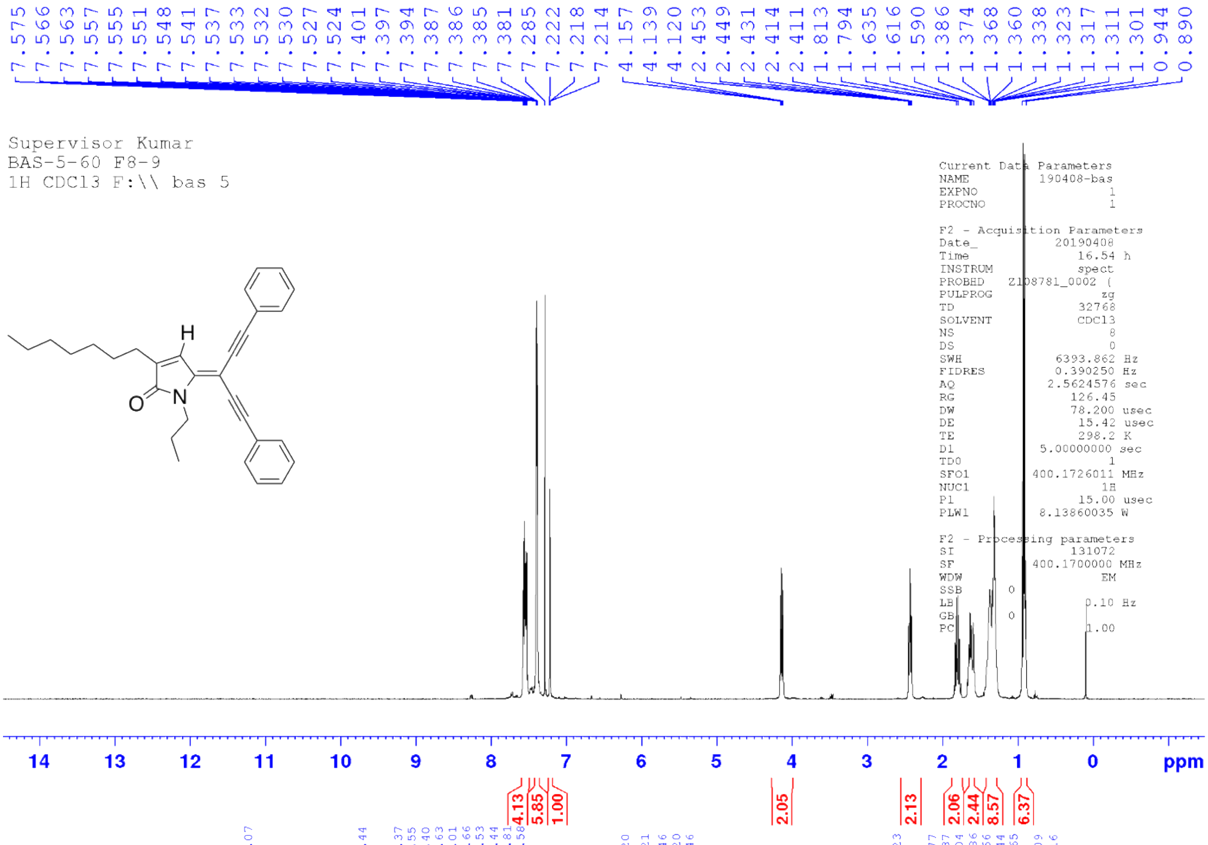


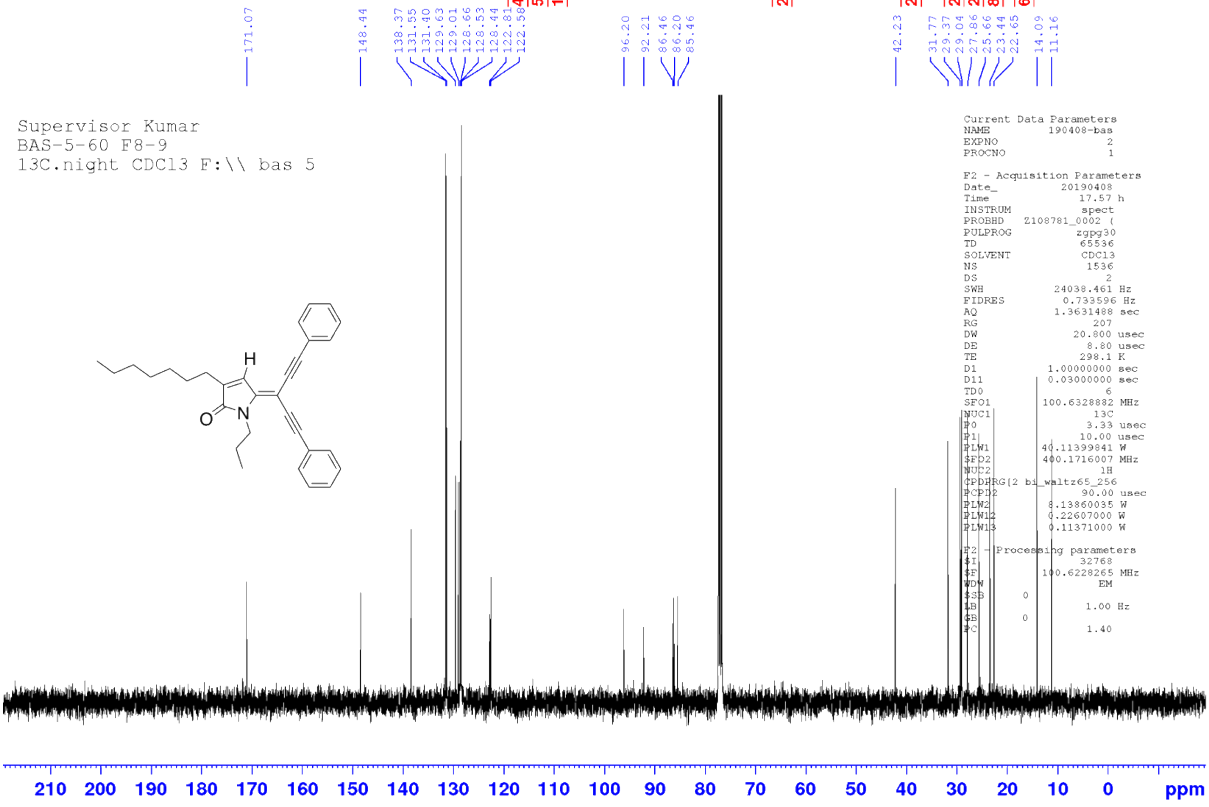

5-(1,5-Diphenylpenta-1,4-diyn-3-ylidene)-3-octyl-1-propyl-1,5-dihydro-2*H*-pyrrol- 2-one (42)

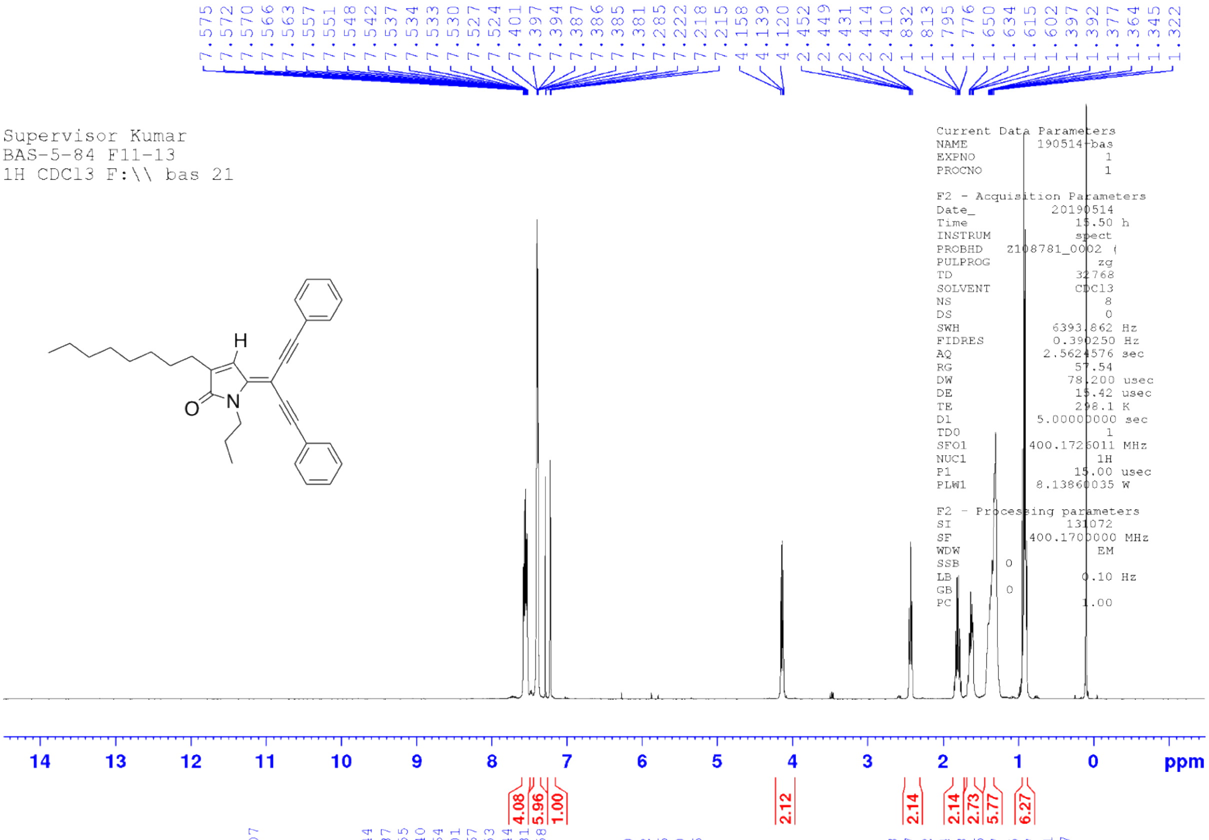


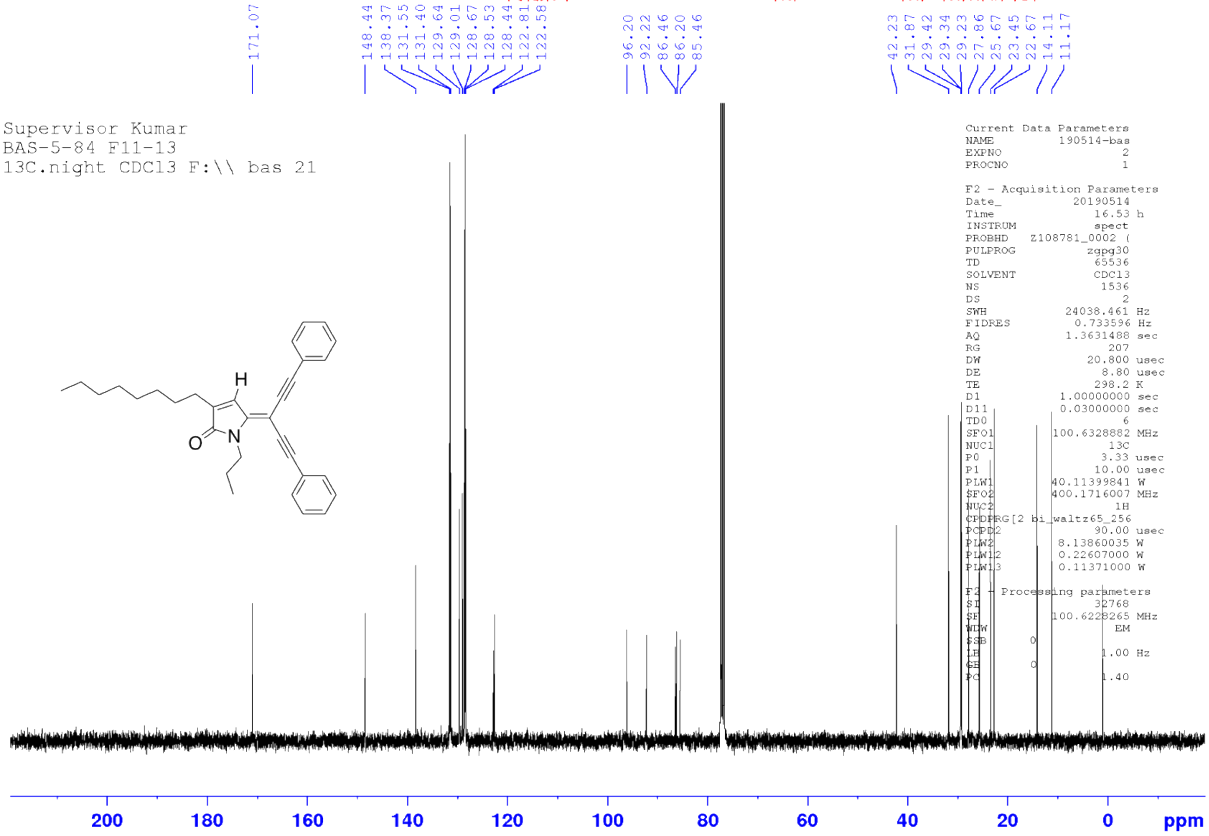

1,3-Dibutyl-5-(1,5-diphenylpenta-1,4-diyn-3-ylidene)-1,5-dihydro-2*H*-pyrrol-2-one (43)

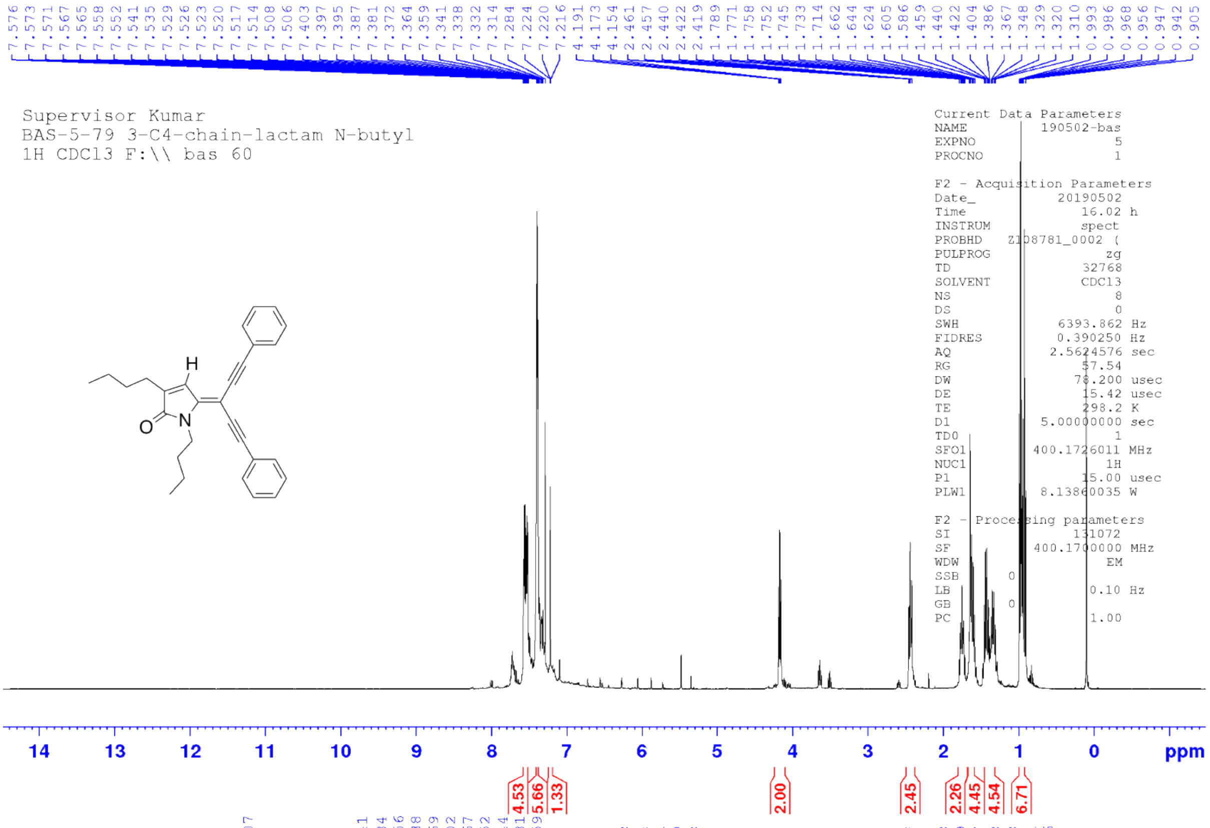

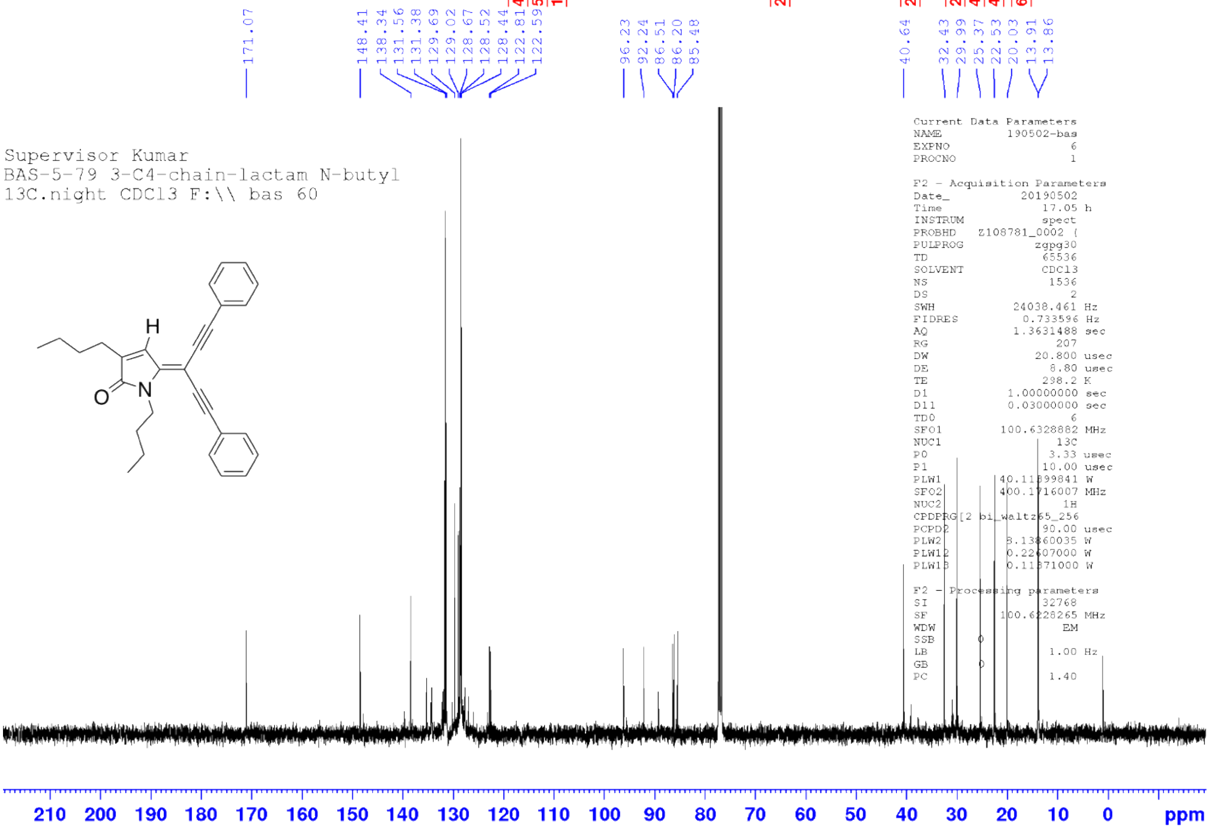

## 3-Butyl-5-(penta-1,4-diyn-3-ylidene)-1-propyl-1,5-dihydro-2*H*-pyrrol-2-one (44)

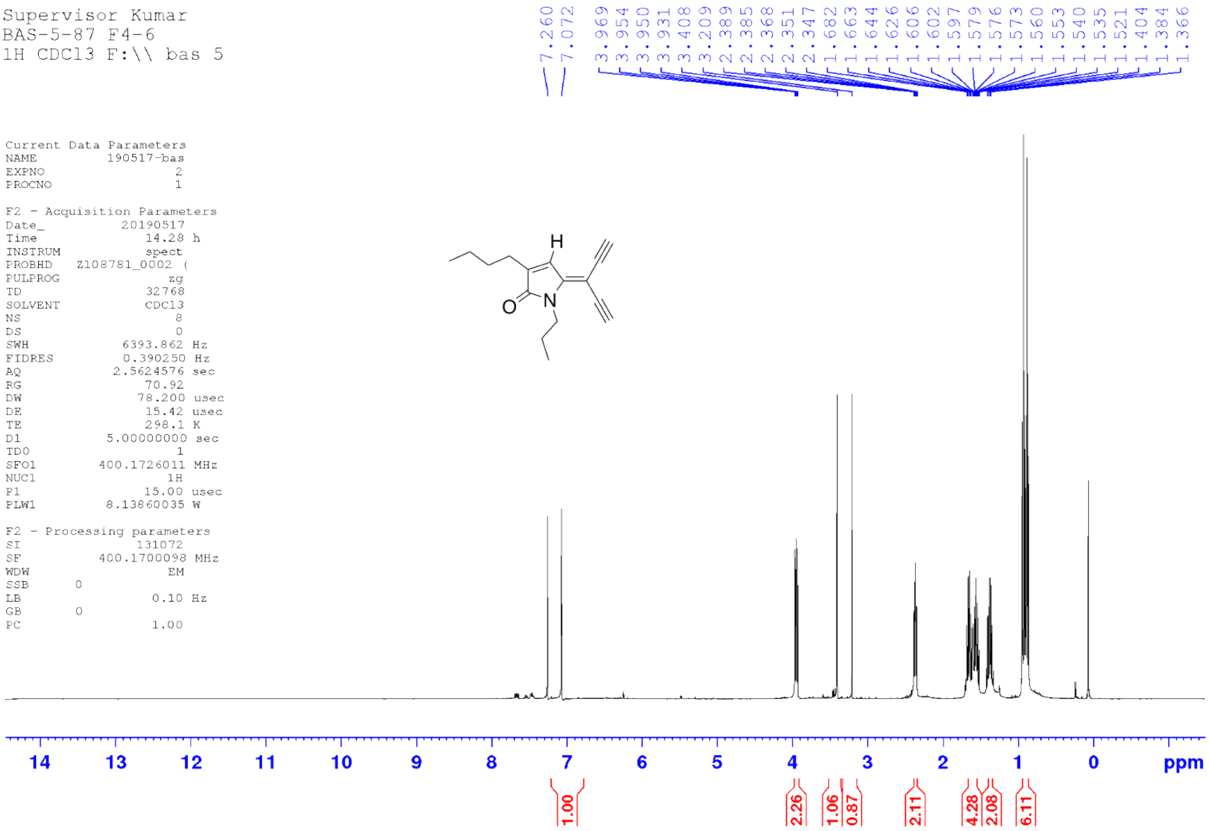


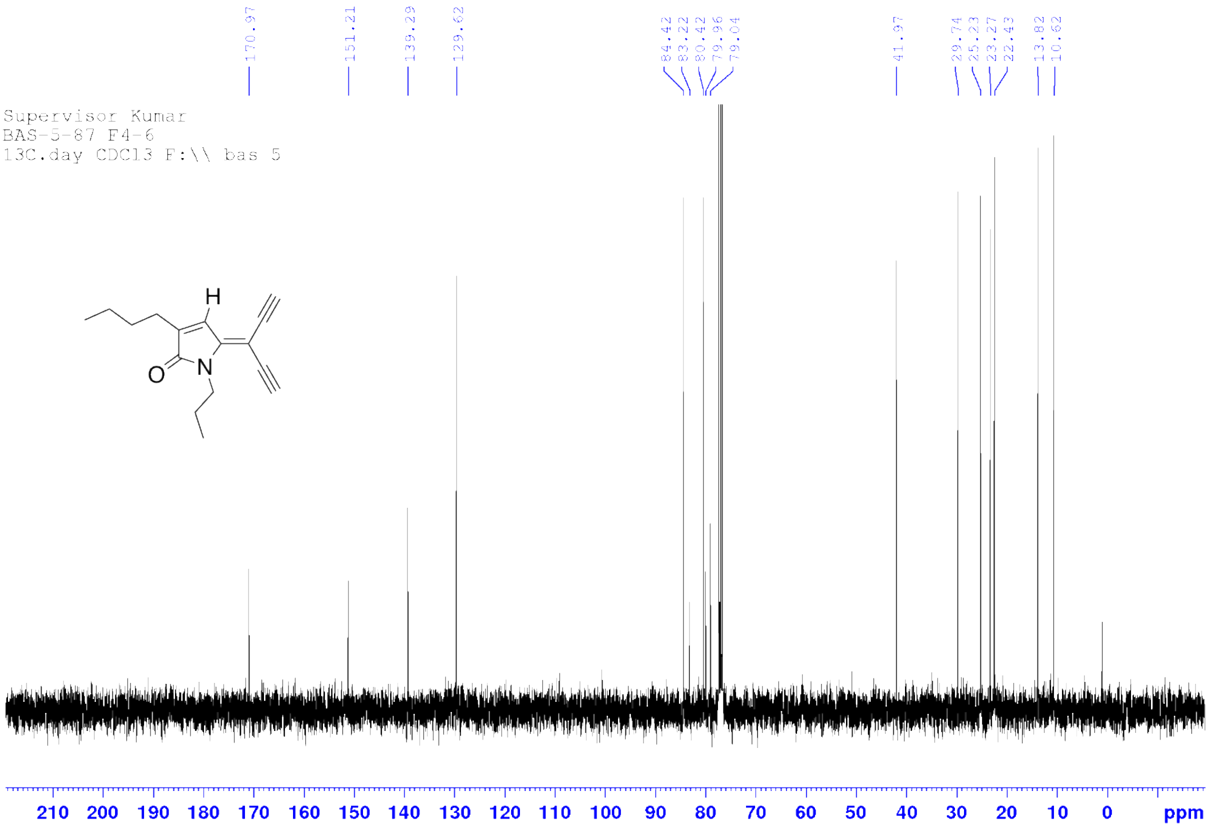

Supplement: Supplementary file 1 [file antibiotics-11-00151-s001.zip › antibiotics-1555965-supplementary.pdf]
